# Surface Ligand Composition of Polyoxovanadate-Alkoxides Influences Hole Transfer from CdSe Quantum Dots

**DOI:** 10.1021/acs.inorgchem.6c01486

**Published:** 2026-10-15

**Authors:** Chari Y. M Peter, Benjamin Hancock, John J. Caruso, Nicholas B. Flemings, Margaret C. Hintz, Todd D. Krauss, Ellen M. Matson

**Affiliations:** † Department of Chemistry, 6927 University of Rochester, Rochester, New York 14627, United States; ‡ Institute of Optics, University of Rochester, Rochester, New York 14627, United States

## Abstract

In this study, we evaluate the impact of the modulation of the surface structure of polyoxovanadate-alkoxides (POV-alkoxides) on their hole transfer capabilities with cadmium selenide quantum dots (CdSe QDs). Initial studies probe the consequences of increasing the carbon chain length of bridging alkoxide ligands, through comparison of efficiencies of hole transfer to methoxide- and ethoxide-capped POV-alkoxides, [V_6_O_7_(OR)_12_]^1–^ (R = CH_3_, CH_2_CH_3_). While [V_6_O_7_(OCH_2_CH_3_)_12_]^1–^ is able to quench the photoluminescence of CdSe QDs, reduced efficiency is observed in comparison to its methoxide congener. This observation indicates that increasing steric bulk at the surface of the POV-alkoxide inhibits interactions of the hole acceptor with the surface of the nanocrystal. Subsequent studies investigate a POV-alkoxide capped with a tridentate ligand containing a pyridine substituent that we hypothesized would promote coordination of the cluster to the surface of the nanocrystal. Steady-state photoluminescence quenching experiments indicate improved hole transfer in comparison to structural controls, while time-correlated single-photon counting photoluminescence decay dynamics confirm an increase in the number of QD-cluster associations. However, slower hole transfer lifetimes per cluster are noted for the pyridine-substituted assembly. Collectively, our experiments provide insight into design criteria for molecular redox mediators that enhance the efficiency of hole transfer from CdSe QDs.

## Graphical abstract

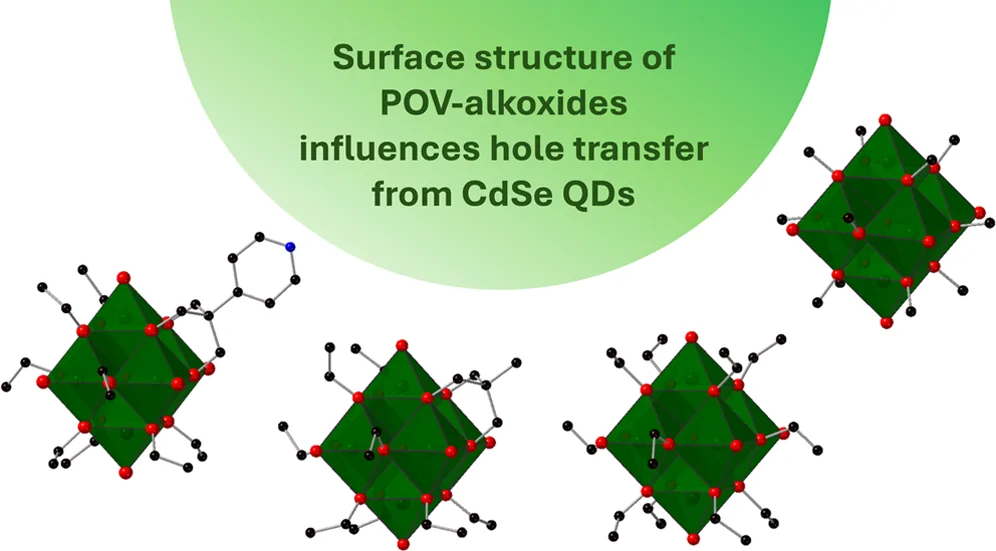

## Introduction

Colloidal semiconductor quantum dots (QDs) have gained attention in recent years as materials for the development of optoelectronic devices, photodetectors, and light-emitting diodes.^1–4^ Interest in these materials stems from their large absorption cross-section, tunable band gaps, and photostability.^5–7^ These same attributes make QDs excellent candidates as light harvesters for photocatalytic reactions. Indeed, over the past decade, this research space has benefited from extensive work, demonstrating the advancement of QD-based colloidal materials for photocatalytic applications. For example, semiconductor QDs, such as CdSe,^8,9^ CdS,^10,11^ ZnSe,^12–14^ CuInS_2_,^15,16^ and InP^17,18^ have proven to be attractive as stable visible-light photosensitizers in comparison to single-molecule photosensitizers such as organic dyes (e.g., Eosin Y), or inorganic complexes (e.g., [Ru­(bpy)_3_]^2+^ and [Ir­(bpy)­(ppy)_2_]­PF_6_).^19–22^ When coupled to a suitable cocatalyst, semiconductor QDs are able to facilitate H_2_ production, yielding an active and robust photocatalytic system.

Our research team is interested in the investigation of cadmium selenide (CdSe) QDs as photosensitizers for solar energy conversion chemistries. Among the wide range of semiconductor nanocrystals available, CdSe is particularly useful due to its strong absorption across much of the visible region and its sufficiently negative conduction band energies.^23^ In addition, the synthesis of CdSe nanocrystals is well-developed, allowing for precise control over particle size, band edge position, and ligand environment.^24–26^ While our work^9,27,28^ and results from others,^29–31^ have demonstrated that CdSe is an effective photosensitizer/photocatalyst for solar fuel production, the kinetics of the extraction of photogenerated holes remain limited by the large effective mass of the hole and surface traps/localization.^32–38^ Hole transfer is particularly challenging for CdSe QDs, due to its shallow valence band (∼+ 1.0 V vs NHE^5^), limiting the thermodynamic driving force to sacrificial hole acceptors.^32,33^ Collectively, these considerations render hole transfer from CdSe QDs the rate-determining step in many photocatalytic systems.

To overcome the kinetic limitations of hole transfer, several studies have been performed to understand how to efficiently extract holes from cadmium chalcogenide QDs. We note that the majority of work has focused on the investigation of cadmium sulfide (CdS) nanocrystals, which have deeper valence bands in comparison to CdSe.^39,40^ Themes that have emerged from these investigations suggest that direct interaction of hole transfer reagents with the surface of the nanocrystal is required for efficient charge transfer. For example, Weiss and co-workers have demonstrated subpicosecond hole transfer from CdS QDs to a phenothiazine acceptor (PTZ) upon covalent attachment of the acceptor to the surface of the nanocrystal through a phenylthiocarbamide linker (PTZ).^41^ Subsequent work from Dukovic and co-workers and Alivisatos and co-workers expands upon this observation through the attachment of phenothiazine derivatives to the surface of the CdS nanocrystal through distinct chemical anchors.^42,43^ Surface coordination of hole transfer reagents has also been demonstrated to be practical: Dukovic and co-workers reported ultrafast hole transfer from CdS QDs to a ruthenium polypyridine water oxidation catalyst when the complex is directly bound to the nanocrystal surface.^44–46^

Covalent attachment of hole scavengers to the surface of CdSe nanocrystals has also proven effective in promoting charge transfer. Alivisatos and co-workers found that attachment of ferrocene derivatives to the surface of CdSe-based core–shell QDs results in hole transfer efficiencies of up to 99%, highlighting the impact of strong surface coordination.^47,48^ Work done by Lian and co-workers has shown that photoexcited CdSe QDs undergo hole transfer to PTZ, which dissociates the exciton leading to the formation of PTZ radicals directly observed by transient absorption spectroscopy.^49^ In addition, surface bound nitroxide radicals such as 4-amino-TEMPO (TEMPO = 2,2,6,6-tetramethylpiperidine-1-oxyl free radical) have been shown to extract holes from CdSe on subpicosecond timescales by McGuire and co-workers, reinforcing that coordinated molecular species play a role in mediating efficient redox processes.^50,51^ In all three examples, the authors propose that coordination of the redox mediator to the nanocrystal surface introduces trap states that are significantly different than those of the native nanocrystal, accelerating the rate of hole transfer through enhanced electronic coupling.

Our research team has been investigating polyoxovanadate-alkoxides (POV-alkoxides) as hole scavengers for CdSe QDs. Our studies have shown that direct attachment to the surface of the nanocrystal is critical for hole transfer. For example, we demonstrated how the incorporation of oxygen-atom vacancies at the surface of POV-alkoxides (e.g., [V_6_O_6_(OCH_3_)_12_]^1–^) improves hole transfer kinetics by promoting specific surface interactions between cluster and QD.^52^ We attribute this efficiency to the presence of the P = O moiety on the ligand, which binds to the O-atom defect site of the POV-alkoxide. In an attempt to develop more general approaches to promoting interactions between CdSe QDs and POV-alkoxides, we have shown that by reducing ligand density at the surface of the nanocrystal, we can increase hole transfer efficiency to a molecular vanadium oxide hole acceptor.^53^ These results show that partial removal of surface ligand density can be used to increase the interactions of the cluster with QDs, thus improving the efficiency of QDs.

An alternative strategy for promoting QD/POV-alkoxide association is through the modification of the organic ligands at the surface of the vanadium oxide assembly. Previous work from our group^54–56^ and others^57,58^ has demonstrated that a range of functional groups can be incorporated at the surface of the Lindqvist ion through the incorporation of a trishydroxymethane (TRIOL) ligand. This tridentate capping ligand replaces three facially positioned bridging alkoxide moieties. Given that the tertiary carbon of the TRIOL ligand can be synthetically modified, providing opportunities to install functional groups at the surface of the cluster complex with minimal impact on the electrochemical properties of the vanadium oxide assembly. In particular, our attention was drawn to a reported POV-alkoxide attached to a pyridine moiety in the 4-position, [V_6_O_7_(OCH_2_CH_3_)_9_TRIOL^Py^]^1–^ (Figure 1). We hypothesized that the pyridine functional group would facilitate coordination of the vanadium oxide assembly to the surface of the CdSe QD based on prior studies that have shown that pyridines are good ligands for these semiconducting nanocrystals.^59–61^

**Figure 1:**
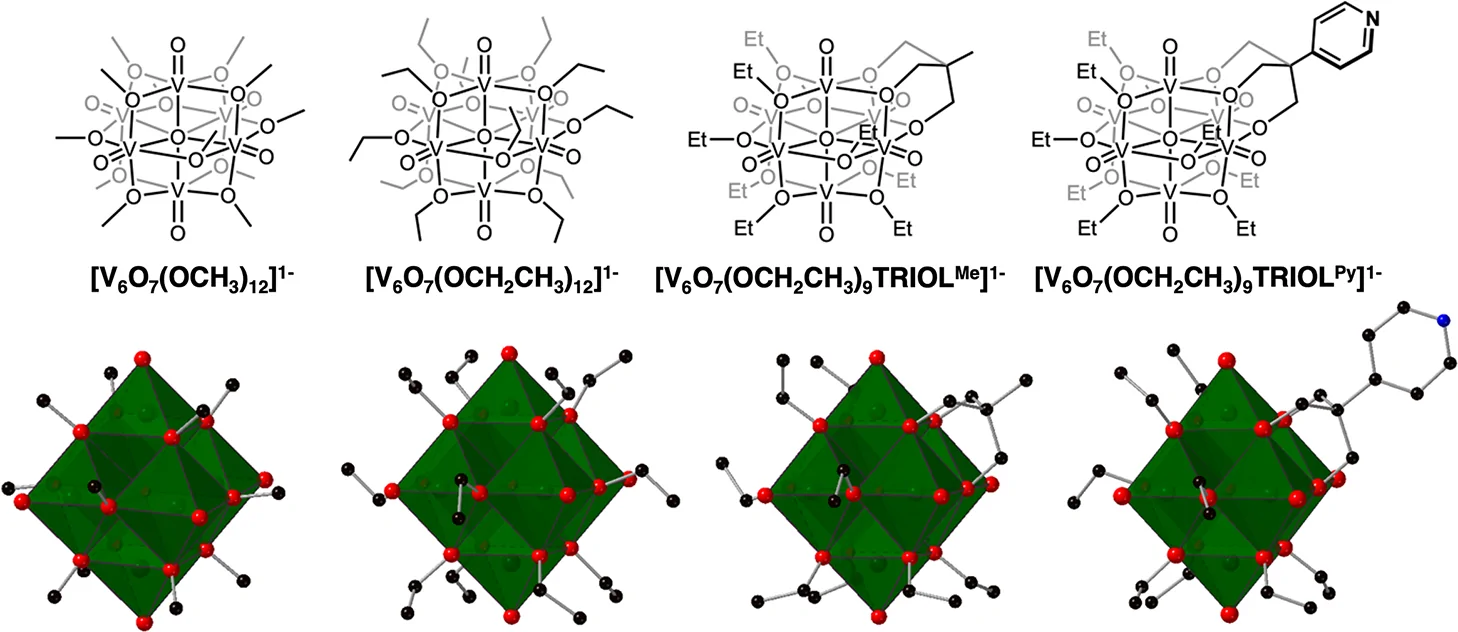
Depiction of the molecular structures of POV-alkoxides investigated in this work. In all cases, the POV-alkoxides of interest are studied in their monoanionic charge state, with a tetrabutylammonium countercation. In this illustration, we have omitted the counterion for clarity.

Herein, we investigate how modifying the identity of surface ligands in POV-alkoxides influences their ability to remove holes from oleic acid (OA) capped CdSe QDs. We first study how steric changes at the cluster surface modulate nanocrystal–cluster interactions through comparison of hole transfer efficiencies with methoxide and ethoxide-capped POV-alkoxides. Introduction of ligand modification to the POV-alkoxide surface allow functional groups to be installed at the surface of the cluster. By comparing hole transfer efficiencies of methyl-capped and pyridine-capped assemblies, we demonstrate how the incorporation of the functional group enhances interfacial association and hole transfer. Steady-state and time-resolved photoluminescence measurements are compared to provide further insight into the nature of QD-cluster contacts, revealing the importance of static mechanisms of charge transfer in the rates of hole transfer reactions. Collectively, these experiments allow us to determine how changes at the cluster surface can directly influence its ability to remove holes from CdSe QDs.

## Experimental Methods

### Chemicals

Chemicals were purchased and used without further purification except where noted. Cadmium (II) oxide (CdO, 99.99%), selenium (Se, 100 mesh, 99.99%), 1-octadecene (ODE, 90%), oleic acid (OA, 90%), trimethyl oxonium tetrafluoroborate (MS, 95%), and tetrabutylammonium borohydride (TBABH_4_, 98%) were purchased from Sigma-Aldrich and used as received. Methylene Chloride (DCM) (Stabilized/Certified ACS, Fisher Chemical) was purchased from Fisher Scientific. Tetrabutylammonium Hexafluorophosphate (TBA PF6, 98%) was purchased from Sigma-Aldrich, recrystallized thrice using hot methanol, and stored under dynamic vacuum for a minimum of two days before use.

#### Synthesis of Oleic Acid (OA)-Capped CdSe Quantum Dots (QDs)

OA CdSe QDs were utilized in these experiments. To a dry, three-neck round-bottom flask, 3 mL of OA, 10 mL of ODE, and 0.514 g of CdO (0.514 g, 4.00 mmol) were added. The flask was placed under an N_2_ atmosphere and heated to 260 °C while stirring at 1100 rpm under reflux. While the solution was heating, the Se precursor was prepared by sonication of 0.68 g of Se in 10 mL ODE until a uniform suspension was formed. Once the Cd precursor became clear and the temperature reached 260 °C, 1 mL Se precursor was rapidly injected. The QDs were allowed to grow for 20 s at 250 °C before thermal quenching with a heat gun (on the cool setting) and a water bath once the temperature reached 180 °C below. In order to remove the unused precursor, the solution was washed with ethanol in a 6:1 (v/v) ratio to QDs followed by centrifugation at 5000 rpm for 15 min. The clear supernatant was then discarded, and the pellet was redispersed in minimal hexane. We repeated the washing once more and stored the final QD solution in 15 mL hexane.

#### Synthesis of [V_6_O_7_(OCH_3_)_12_]^1–^

Cluster was prepared according to previous literature.^62^

#### Synthesis of [V_6_O_7_(OCH_2_CH_3_)_12_]^1–^

Cluster was prepared according to previous literature.^54^

#### Synthesis of [V_6_O_7_(OCH_2_CH_3_)_9_TRIOL^Me^]^1–^

Cluster was prepared according to previous literature.^54^

#### Synthesis of [V_6_O_7_(OCH_2_CH_3_)_9_TRIOL^Py^]^1–^

Cluster was prepared according to previous literature.^54^

#### OA-CdSe + Meerwein’s Salt Quantitative ^1^H NMR Spectroscopy

All ^1^H NMR samples were prepared outside of a glovebox. ^1^H NMR samples were prepared by first drying aliquots from a QD stock that would yield 100 μM samples under an air stream. A ferrocene internal standard solution was prepared by dissolving 5 mg of ferrocene in 500 μL of toluene-d_8_. The 4 mM stock solutions of trimethyl oxonium tetrafluoroborate (Meerwein’s Salt, MS) in DCM were prepared inside a nitrogen-filled glovebox. To the dried QDs, an appropriate volume of DCM and the MS stock solution was added such that the total volume reached 600 μL for all samples (0, 1, 5, 10, 15, 20 equivalents MS). All samples were left to sit for 1 h in the DCM suspensions, then dried under an airstream and resuspended in 590 μL toluene-d_8,_ and 10 μL of the ferrocene internal standard solution was added to each NMR tube. All ^1^H NMR spectra were collected on a Bruker 400 MHz spectrometer. Following ^1^H NMR spectrum collection, quantification of bound oleic acid and free methyl oleate was obtained via peak fitting in MestReNova.^63^

### Photoluminescence Quenching

QD samples were placed in a quartz cuvette with a 1 cm path length for all absorption and emission characterization. A PerkinElmer Lambda 950 UV/vis/NIR spectrophotometer was used to record all absorbance spectra. Following all syntheses, the OA-CdSe QD concentration was calculated using the first excitonic absorbance transition as described by Yu et al.^3^ Photoluminescence (PL) spectra were measured with a Horiba FluoroMax + SpectroFluorometer. OA-CdSe QDs were treated with 20 equivalents of MS in dichloromethane. This was then followed by the addition of varying equivalents of cluster in each fluorometer cuvette.

### Time-Correlated Single Photon Counting

Ensemble TCSPC measurements were acquired using a home-built optical setup featuring an inverted microscope (Nikon, TE-200U) with a 10× air objective (Nikon, NA 0.3) and a 488 nm dichroic mirror (Semrock, Inc.). Samples, which had been transferred to quartz cuvettes with 1 mm thick windows, were photoexcited by a defocused laser beam provided by a pulsed (5 MHz) supercontinuum source (FYLA Iceblink), with 488 nm excitation selected through bandpass and short-pass filters. Power-dependent studies were performed to ensure that excitation remained in the single-exciton regime and the same excitation power used for all samples. Photoluminescence (PL) emission was collected by a single-photon avalanche diode detector (Micro Photon Devices, PDM Series, 50 μm active sensing area diameter) with a fast-timing resolution (56 ps). All components of the TCSPC setup were incorporated in the PicoHarp 300 module (PicoQuant). The instrument response function was collected from residual scattering of the light from the pulsed laser. To collect lifetime decay curves, 488 nm long-pass filters were placed in the emission pathway to block scattered excitation light. Data collection was halted once a 1000 count maximum was reached and the count rate was kept below 1% the laser repetition rate. Decay curves corresponding to OA-CdSe QDs + *n* eq MS without the quencher were first fit to a triple exponential decay to establish the dynamics before PL quenching was initiated. Subsequent decay curves corresponding to OA-CdSe QDs + *n* eq MS with the quencher were fit according to the lifetime parameters calculated for the prequenching sample and an additional, floating parameter used to examine the quenching process.

### Transient Absorption Spectroscopy

We performed ultrafast transient absorption (TA) spectroscopy to probe the excited-state dynamics. The measurements were performed using an ultrafast laser system in a pump–probe configuration. Femtosecond pulses (277 fs) from a 1035 nm diode-pumped fiber laser (Coherent, Monaco IR) at 500 kHz were introduced into an optical parametric amplifier (Coherent, Opera-NOPA). The resulting signal pulses (≈50 fs, 500 kHz) were tuned to the desired wavelength (535 nm) and used as a pump. The probe beam was generated using a YAG crystal pumped by the fundamental 1035 nm from Monaco IR. The reference beam was split from the probe before the sample and used to correct the probe for pulse-to-pulse fluctuations. A motorized delay stage was used to control the optical time delay between the pump and probe pulses. Samples were placed in a 2 mm path length quartz cuvette and stirred to avoid laser-induced signal artifacts. The optical density of CdSe QDs was 0.1 at the pump wavelength of 535 nm. The average number of excitons absorbed per QD, ⟨*N*_0_⟩, was kept below one for all measurements.

### Hole Transfer Efficiencies Calculations

Hole transfer efficiency derived from PL lifetimes can be calculated by taking the ratio of the integrated area under the time-resolved PL decay curves with and without the cluster according to^2^

1 $$E_{HT}^{lifetimes}=1-\frac{\int PL\left(QDs+MS+V_{6}O_{7}^{1-}\right)}{\int PL\left(QDs+MS\right)}$$

And the hole transfer efficiency based on steady-state (quenching) is defined as

2 $$E_{HT}^{quenching}=1-\frac{I}{I_{0}}$$

### PL Decay Dynamics Analysis

Intrinsic QD dynamics were obtained by fitting the kinetics of QDs + *n* eq MS (*n* = 20) without clusters to yield the amplitudes Ai and time constants τQD_*i*_ (τQD*i* = 1/*k*QD_*i*_). The kinetics of the QDs + *n* eq MS with various [V_6_O_7_(OR)_12_]^1−^ cluster concentrations were globally fit, holding the amplitudes Ai and rate constants *k*QD_*i*_ of the intrinsic electron relaxation constant. To account for the HT process (assumed to be monoexponential), the number of acceptors m was allowed to vary, while the rate constant kHTj was held constant, as it denotes the rate for a 1:1 cluster to QD + *n* eq MS system. The intrinsic QD + *n* eq MS dynamics and the HT process are treated independently, leaving ∑*iA*_*i*_ = 1. The lifetimes reported are amplitude-averaged lifetimes $\tau_{QD}=\frac{\sum_{i}A_{i}\tau_{QDi}}{\sum_{i}A_{i}}$ , appropriate for the Stern–Volmer analysis performed.^64,65^

### Number of Molecular Acceptors Estimation

We estimate the number of molecular acceptors [V_6_O_7_(OR)_12_]^1−^ interacting with the QDs by fitting the PL lifetime data to a model proposed by Tachiya et al.^66^ This model assumes a Poissonian distribution of quencher per chromophore given by

3 $$f_{n}=\frac{m^{n}}{n!}e^{-m}$$

where *f*_*n*_ is the probability of a given QD being quenched by *n* [V_6_O_7_(OR)_12_]^1−^ molecules and *m* = ⟨*n*⟩.^52,53,65,66^ The total rate of hole transfer is the product of the effective number of POV-alkoxides per QD (*n*) and the hole transfer rate for one cluster per QD

4 $$k_{HT-total}=nk_{HT}$$

Therefore, the PL decay of excited QDs as a function of time is represented by the equation

5 $$I\left(t\right)=I_{0}\left(\sum_{i}A_{i}e^{-k_{QD_{i}}t}\right)\left(\sum_{n=0}^{\infty }f_{n}e^{-nk_{HT}t}\right)$$

*I*_0_ is a normalization constant corresponding to the initial concentration of excited QDs and *A*_*i*_ is the amplitude of the *i*th component of the QD PL decay without a cluster. It can be mathematically demonstrated that the sum corresponding to the hole transfer process (right sum in equation 3) can be simplified to^66^

6 $$\left(t\right)=I_{0}\left(\sum_{i}A_{i}e^{-k_{QD_{i}}t}\right)\left[e^{\left(-m+me^{-k_{HT}t}\right)}\right]$$

An iterative reconvolution process was utilized to produce fits to the prior equations, where

7 $$I\left(t\right)=\left[I_{Actual}\left(t\right)\times IRF\left(t-\Delta t\right)\right]+B$$

*I*_Actual_ corresponds to the QD PL decay before convolution with the IRF to produce the measured signal and therefore contains the desired photophysics. This was first utilized to obtain the intrinsic QD decay dynamics, and subsequently within the global fit according to eq 4 to estimate the number of molecular acceptors.

## Results and Discussion

As described in the introduction of this work, we hypothesized that modulation of hole transfer from CdSe QDs to POV-alkoxides would be possible through modifications to surface ligands of the molecular vanadium oxide assembly. Specifically, we were interested in the incorporation of a molecular “anchor” that would promote direct coordination of the Lindqvist ion to the surface of the nanocrystal. Toward this goal, we selected [V_6_O_7_(OCH_2_CH_3_)_9_TRIOL^Py^]^1−^ for study, as this cluster contains a pyridine functional group that we proposed would associate with the nanocrystal surface.

Our interest in investigating photoluminescence (PL) quenching of CdSe QDs by [V_6_O_7_(OCH_2_CH_3_)_9_TRIOL^Py^]^1−^ introduces a number of differences to the surface structure of the vanadium oxide assembly from that of the previously investigated methoxide-capped POV-alkoxide, [V_6_O_7_(OCH_3_)_12_]^1–^ (see Figure 1 for comparative structures).^53^ For example, the nine bridging ethoxide ligands will likely reduce the extent of interactions of the POV-alkoxide and CdSe QD. Additionally, the change in molecular symmetry of the POV-alkoxide upon incorporation of the TRIOL capping ligand will have an impact on the molecular orbitals of the assembly. Both of these factors could influence the efficiency of charge transfer. To rule out these contributions, we also present hole transfer studies with [V_6_O_7_(OCH_2_CH_3_)_12_]^1–^ (a control for the impact of bridging alkoxide ligand length) and [V_6_O_7_(OCH_2_CH_3_)_9_TRIOL^Me^]^1–^ (a control for the reduction in symmetry of the POV-alkoxide).

Syntheses and characterization of POV-alkoxides were accomplished according to literature precedent.^54,67–69^ In the original report describing the synthesis and characterization of these POV-alkoxides, electrochemical data were reported in acetonitrile (MeCN). However, our studies on hole transfer from CdSe QDs to POV-alkoxides have all been performed in dichloromethane (DCM), as both cluster and nanocrystal are soluble in this solvent. To confirm that similar electrochemical properties are observed for the POV-alkoxide in DCM, the cyclic voltammograms (CVs) of [V_6_O_7_(OR)_12_]^1–^ (R = CH_3_, CH_2_CH_3_), and [V_6_O_7_(OCH_2_CH_3_)_9_TRIOL^R^]^1–^ (R = Me, Py) were collected in this solvent (0.1 tetrabutylammonium hexafluorophosphate, TBAPF_6_, used as the supporting electrolyte). The CVs of these complexes are displayed in Figure 2, with corresponding redox couples and reduction potentials summarized in Table 1. We note that the CVs of all four POV-alkoxides are similar (Figure 2); only minor changes in reduction potential are observed, indicating that all three POV-alkoxides have similar driving forces for hole transfer from CdSe QDs, despite changes to their overall surface structure.

**Figure 2:**
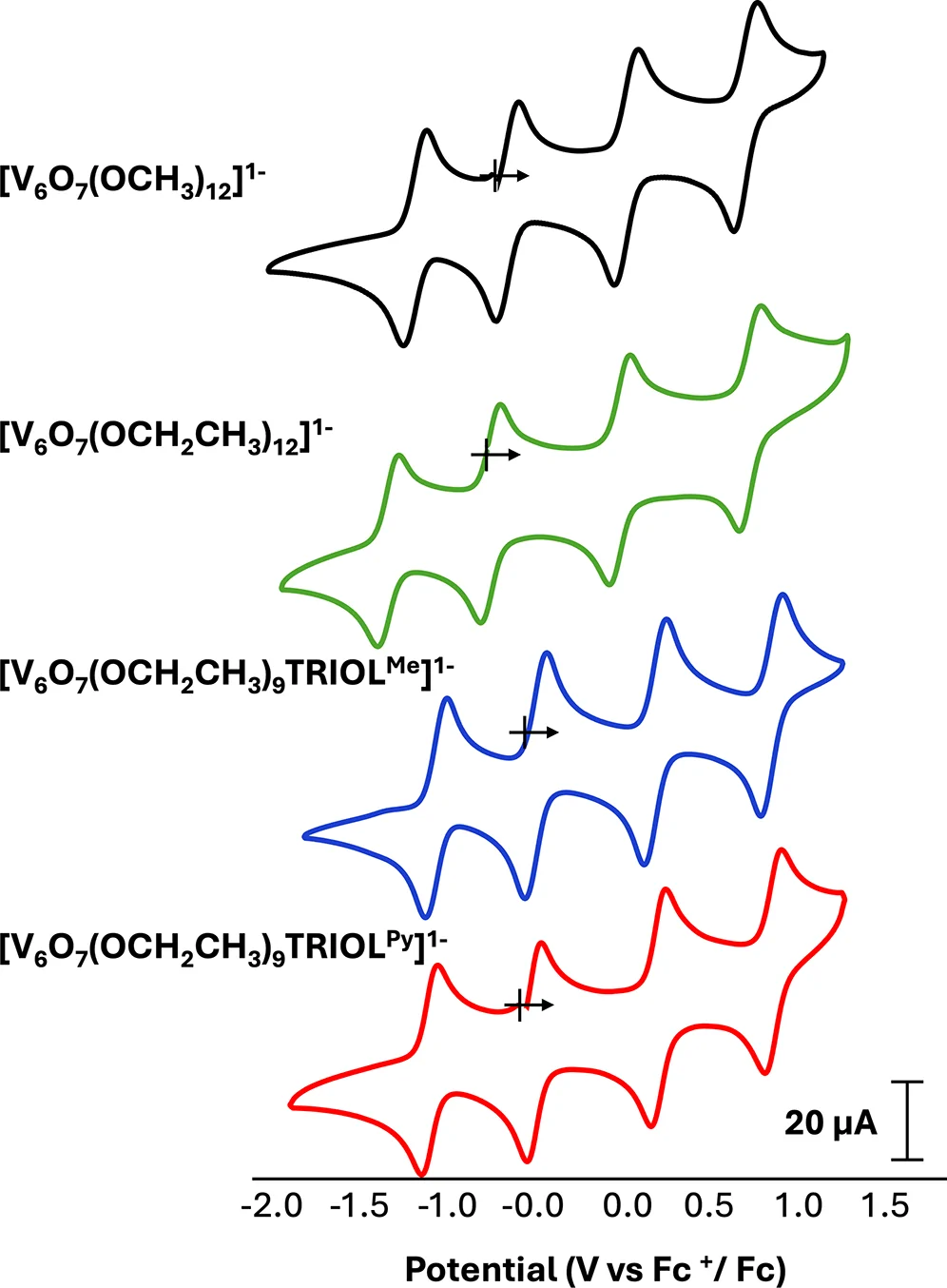
CVs of **[V**_**6**_ **O**_**7**_(**OR**)_**12**_ **]** ^**1**–^ (R = CH_3_, black; CH_2_CH_3_, green) and **[V**_**6**_ **O**_**7**_(**OCH**_**2**_ **CH**_**3**_)_**9**_ **TRIOL** ^**R**^ **]** ^**1**–^ (R = Me, dark blue; Pyr, red) in DCM. Electrochemical measurements were collected using 1 mM solutions of the POV-alkoxide in DCM with 0.1 M TBAPF_6_ as the supporting electrolyte (scan rate = 100 mV s^–1^). Arrows indicate the open-circuit potential of the experiment, confirming all clusters are isolated in the monoanionic charge state. Redox potentials are reported in Table 1.

**Table 1:** Electrochemical Parameters of **[V**_**6**_ **O**_**7**_(**OR**)_**12**_ **]** ^**1**–^ (R = CH_3_; CH_2_CH_3_) and **[V**_**6**_ **O**_**7**_(**OCH**_**2**_ **CH**_**3**_)_**9**_ **TRIOL** ^**R**^ **]** ^**1**–^ (R = Me; Pyr) Measured in DCM With 0.1 M TBAPF_6_ as the Supporting Electrolyte^a^ ^b^

compound	V^V^V^IV^_5_/V^IV^_6_	V^V^_2_V^IV^_4_/V^V^V^IV^_5_	V^V^_3_V^IV^_3_/V^V^_2_V^IV^_4_	V^V^_4_V^IV^_2_/V^V^_3_V^IV^_3_
**[V**_**6**_ **O**_**7**_(**OCH**_**3**_)_**12**_ **]** ^**1**–^	–0.94 V	–0.44 V	+0.20 V	+0.85 V
**[V**_**6**_ **O**_**7**_(**OCH**_**2**_ **CH**_**3**_)_**12**_ **]** ^**1**–^	–1.17 V	–0.63 V	+0.05 V	+0.74 V
**[V**_**6**_ **O**_**7**_(**OCH**_**2**_ **CH**_**3**_)_**9**_ **TRIOL** ^**Me**^ **]** ^**1**–^	–1.03 V	–0.48 V	+0.18 V	+0.83 V
**[V**_**6**_ **O**_**7**_(**OCH**_**2**_ **CH**_**3**_)_**9**_ **TRIOL** ^**Pyr**^ **]** ^**1**–^	–1.02 V	–0.46 V	+0.21 V	+0.80 V

a All Redox Events are Reported vs. Fc^+/0^ Reference.

b Shaded column corresponds to the redox couple of interest in this study; hole transfer from CdSe QDs results in oxidation of the POV-alkoxide in its monoanionic charge state (oxidation state distribution: V^V^V^IV^_5_) and formation of the neutral assembly (oxidation state distribution: V^V^_2_V^IV^_4_).

To study the impact of modifications to the POV-alkoxide surface, we explored the interactions of the assemblies with both natively synthesized OA-capped CdSe QDs, as well as QDs treated with 20 equivalents of trimethyloxonium tetrafluoroborate (Meerwein’s Salt, MS). Our team has previously reported that partial stripping of OA capping ligands is achieved with the use of MS,^53^ building upon seminal work that has demonstrated the use of this reagent for complete removal of surface ligands from a range of nanocrystals.^70–72^ Upon the addition of equivalents of MS ranging from 1 to 20 to OA-CdSe, ^1^H NMR spectroscopy reveals the decrease in the intensity of the signal corresponding to bound OA at 5.6 ppm, with concomitant formation of free methyl oleate at 5.4 ppm proportional to the amount of MS added. The postsynthetic modification of the nanocrystal surface improves access to redox mediators, which is correlated to enhanced charge transfer, while retaining the colloidal stability of the QD in nonpolar organic solvent (e.g., DCM).

Addition of [V_6_O_7_(OCH_2_CH_3_)_12_]^1–^ to OA-capped CdSe QDs (QY: 12.54% ± 1.2) in dichloromethane results in limited quenching of PL based on steady-state analysis (Figure S1). This result is unsurprising, based on our prior results with the POV-methoxide assembly where only ∼20% of PL is quenched after the addition of 20 equivalents of [V_6_O_7_(OCH_3_)_12_]^1–^. However, upon the addition of [V_6_O_7_(OCH_2_CH_3_)_12_]^1–^ to OA-CdSe QDs treated with 20 equiv of MS (QY: 23.7% ± 4.3), steady-state PL measurements show increased quenching (Figure 3). As the concentration of POV-ethoxide added increases, the extent of PL quenching also increases (Figure 3A,C). At 20 equivalents of cluster, the PL intensity is reduced by ∼47%, indicating that interfacial HT to the POV-alkoxide remains possible despite the increase in bridging alkoxide ligand length. However, we note that the extent of charge transfer observed is substantially reduced relative to that observed upon addition of the POV-methoxide to OA-CdSe QDs treated with 20 equiv of MS (Figure 3D,F); in these experiments, ∼75% quenching is observed upon addition of 20 equiv of cluster.

**Figure 3:**
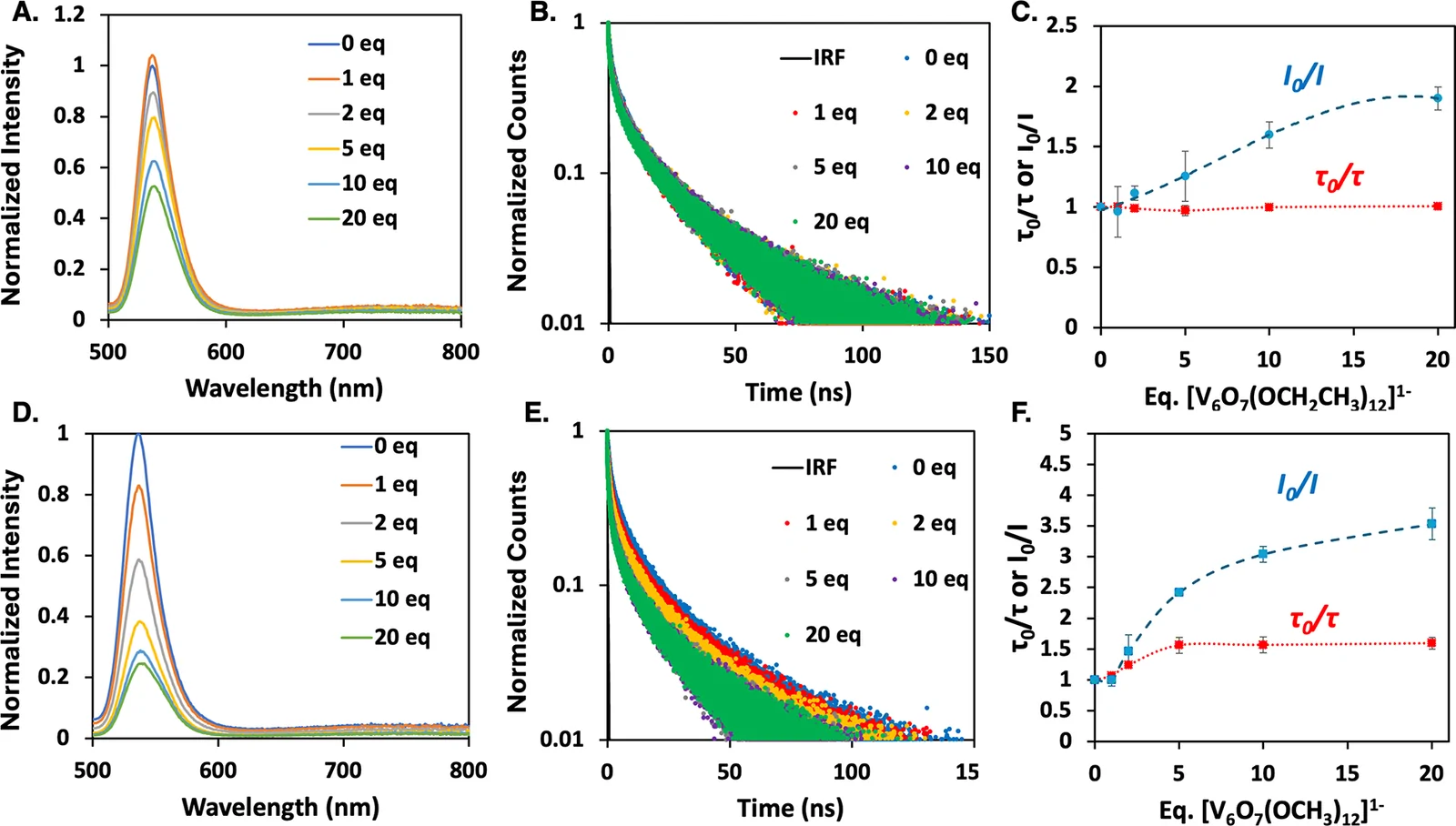
(A) Normalized PL intensity and (B) normalized PL kinetics of OA-capped CdSe QDs +20 equiv of MS with an increasing number of [V_6_O_7_(OCH_2_CH_3_)_12_]^1–^ equivalents. (C) Steady-state (blue) and time-resolved (red) PL quenching. The blue and red dotted lines are a guide to the eye. τ_0_ is the amplitude-weighted average PL lifetime of CdSe QDs treated with 20 equiv of MS, where τ is the lifetime of the same nanocrystals following addition of [V_6_O_7_(OCH_2_CH_3_)_12_]^1–^. (D) Normalized PL intensity and (E) normalized PL kinetics of OA-capped CdSe QDs +20 equiv of MS with an increasing number of [V_6_O_7_(OCH_3_)_12_]^1–^ equivalents. (F) Steady-state (blue) and time-resolved (red) PL quenching. The blue and red dotted lines are a guide to the eye. τ_0_ is the amplitude-weighted average PL lifetime of CdSe QDs treated with 20 equiv of MS, and τ is the amplitude-weighted average lifetime of the same nanocrystals with added [V_6_O_7_(OCH_3_)_12_]^1–^. The lifetimes in (C,F) are extracted from separate triple exponential tail fits independent of our analysis with the Tachiya model. Short timescales for TCSPC traces are shown in Figure S5.

The QD PL intensity (*I*) was plotted as a function of the increasing cluster concentration, according to *I*_0_/*I* = 1 + *K*_sv_[POV-alkoxide], where *K*_sv_ is the Stern–Volmer quenching constant, and *I*_0_ is the original PL intensity in the absence of clusters. *K*_sv_ for the POV-methoxide (1.5 × 10^5^ M ^1–^) is an order of magnitude larger than that observed in the POV-ethoxide (4.6× 10^4^ M^–1^). The difference in steady-state PL quenching between the POV-methoxide and -ethoxide assemblies indicates that increasing the chain length of bridging alkoxide ligands flanking the surface of the Lindqvist core reduces the extent of interactions of the cluster with CdSe QD.

In our prior work, the nature of interactions between OA-capped CdSe nanocrystals treated with MS with the POV-methoxide cluster [V_6_O_7_(OCH_3_)_12_]^1–^ was also interrogated using time-resolved fluorescence quenching studies to understand the nature of the interactions between the cluster and the QD. The PL decay dynamics of the CdSe QDs were evaluated under conditions identical to those presented in the steady-state PL quenching experiments (see Experimental Section for details). Here, we apply the same analysis to the quenching of the PL of the CdSe QD by [V_6_O_7_(OCH_2_CH_3_)_12_]^1–^ (see Experimental Section for additional details). Although there is a significant decrease observed in steady-state PL intensity upon the addition of cluster equivalents, the excited-state lifetimes remain largely unchanged as additional cluster equivalents are added to the sample (Figure 3C). This result suggests that the decrease in emission intensity is a result of predominantly static-like quenching of the POV-ethoxide bound to the nanocrystal surface (exhibited as quenching faster than the IRF), as opposed to a mixture of quenching faster than the IRF and dynamic-like quenching observed with the POV-methoxide cluster.

To quantitatively evaluate the extent of QD-cluster associations, we analyzed the time-resolved PL traces using a method described by Tachiya and co-workers.^66^ In this approach, the distribution of acceptors interacting with each QD is treated statistically, following a Poissonian distribution. The time-resolved PL decay can thus be described in terms of an average amount of acceptors (*m*), which corresponds to the effective number of POV-alkoxides associating with a QD. With the addition of 20 equiv of POV-ethoxide, the number of associated assemblies is found to be ∼0.1 per nanocrystal (Table S1). This value is lower than POV-methoxide (*m* ∼ 0.6) (Table S2) under analogous reaction conditions, consistent with the reduced PL quenching observed in the steady-state experiments for the ethoxide-capped assembly. This analysis confirms that while charge transfer remains accessible, the increased length of the bridging alkoxide ligands flanking the surface of the POV-alkoxide limits the number of close dynamic-like QD-cluster interactions possible in this system.

It is worth commenting on how the kinetics of hole transfer change as a function of the bridging alkoxide ligand in the POV-alkoxides studied above. In the case of [V_6_O_7_(OCH_2_CH_3_)_12_]^1–^, the hole transfer lifetime was determined to be 0.07 ± 0.02 ns (Table S1). This value is significantly faster than the lifetime of the CdSe QD treated with 20 equiv of MS in the presence of the POV-methoxide (τ_HT_ = 0.30 ± 0.04 ns). We propose the discrepancies in hole transfer kinetics are a result of the distinct mechanisms of charge transfer operative in these two systems. Evaluation of the time-resolved PL decay traces of the CdSe QD with POV-methoxide and -ethoxide reveals that the shorter-chain vanadium oxide assembly has a larger component of dynamic-like interactions with the nanocrystal surface (Figure 3E). While on average, the number of QD-cluster associations is higher, the small size of the hole acceptor promotes increased diffusion of the assembly to and from the surface of the nanocrystal. In contrast, charge transfer to the POV-ethoxide is almost static-like in nature (Figure 3B). This difference is particularly pronounced when comparing the hole transfer efficiencies determined from steady-state and TRPL for both clusters (Figure S7), with POV-methoxide demonstrating a much larger relative weight of TRPL/steady-state quenching efficiency (0.58 ± 0.06) compared to the POV-ethoxide (0.15 ± 0.13), while the percentage of QDs with strongly interacting cluster (difference between efficiencies calculated from steady-state and TRPL) is similar at 32 ± 6% for POV-methoxide and 40 ± 7% for POV-ethoxide. We note that in our model, the overall hole transfer rate is the product of the average number of acceptors per QD (*m*) times the rate per acceptor,^66^ indicating that a combination of intersecting factors are important when designing hole scavengers for cadmium chalcogenide photosensitizers.

While this study constitutes the first example of the investigation of the influence of POV-alkoxide ligand length on efficiency of hole transfer, we note that our team has previously investigated how the physicochemical properties and reactivity of organosubstituted vanadium oxide assemblies change as a function of the identity of the bridging alkoxide. As shown in Figures 1 and 2, redox potentials and molecular structure remain relatively constant across the two complexes. Additionally, rate constants of heterogeneous electron transfer and diffusion coefficients are minimally influenced by changing the surface ligands from methoxide to ethoxide.^62,67^ The results presented here suggest that access to the surface vanadyl sites is required for efficient hole transfer; as the alkoxide ligand length increases, the vanadyl sites are sterically hindered, thus suppressing charge transfer. The importance of the accessibility of the metal oxide core is supported by prior work from Watson and Banerjee; in this work, the authors demonstrate that the efficiency of excited-state charge transfer is strongly dependent on the close, interfacial contact between the CdSe QD and β-Pb_0.33_V_2_O_5_ nanowires.^73^

To evaluate whether the incorporation of a Lewis basic functional group at the surface of the vanadium oxide assembly can further modulate hole transfer, we next examined a series of POV-ethoxides capped with TRIOL ligands (TRIOL^R^, R = Me, Py), namely [V_6_O_7_(OCH_2_CH_3_)_9_TRIOL^Me^]^1–^ and [V_6_O_7_(OCH_2_CH_3_)_9_TRIOL^Py^]^1–^ (Figure 1). These POV-alkoxides provide a platform for systematically investigating the impact of incorporation of an “anchor” at the surface of the vanadium oxide assembly. Evaluation of steady-state PL experiments with natively synthesized OA-capped CdSe QDs reveals minimal quenching (Figures S2 and S3), re-emphasizing the importance of surface accessible sites to facilitate the formation of cluster/nanocrystal interactions. All measurements discussed in the remainder of this work were conducted using OA-capped CdSe QDs treated with 20 equiv of MS.

Addition of increasing equivalents of [V_6_O_7_(OCH_2_CH_3_)_9_TRIOL^Me^]^1–^ to OA-capped CdSe QDs treated with 20 equiv of MS results in quenching of the PL of the QD (Figure 4A). The intensity of the PL decreases similarly to the POV-ethoxide with an increase in the cluster/QD ratio (1:1 → 20:1). In the case of the largest amount of cluster equivalents studied in this work, the PL of the POV-ethoxide, [V_6_O_7_(OCH_2_CH_3_)_12_]^1–^ presented above quenches ∼47%, compared to ∼53% with [V_6_O_7_(OCH_2_CH_3_)_9_TRIOL^Me^]^1–^. The comparable amount of PL quenching of CdSe QDs observed in the presence of both [V_6_O_7_(OCH_2_CH_3_)_12_]^1–^ and [V_6_O_7_(OCH_2_CH_3_)_9_TRIOL^Me^]^1–^ indicates that the change in molecular symmetry upon addition of the TRIOL ligand does not dramatically influence HT. This comparison serves as an important control, given the potential importance of the shape and diffusivity of molecular orbitals in charge transfer processes.

**Figure 4:**
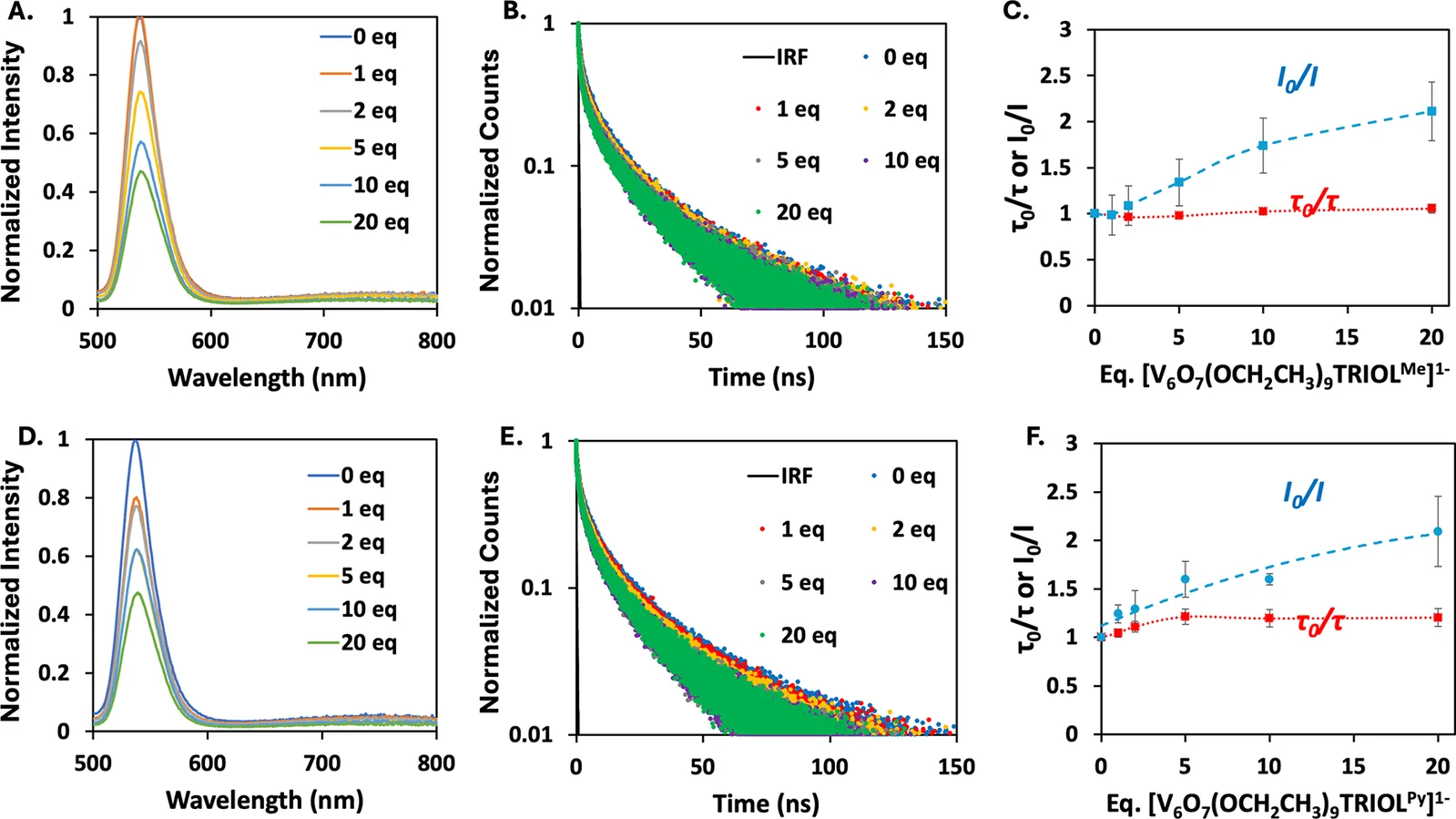
(A) Normalized PL intensity and (B) normalized PL kinetics of OA-capped CdSe QDs +20 equiv of MS with an increasing number of [V_6_O_7_(OCH_2_CH_3_)_9_TRIOL^Me^]^1–^ equivalents. (C) Steady-state (blue) and time-resolved (red) PL quenching. The blue and red dotted lines are a guide to the eye. τ_0_ is the amplitude-weighted average PL lifetime of just the QDs + MS, and τ is the lifetime with added [V_6_O_7_(OCH_2_CH_3_)_9_TRIOL^Me^]^1–^. (D) Normalized PL intensity and (E) normalized PL kinetics of OA-capped CdSe QDs +20 equiv of MS with an increasing number of [V_6_O_7_(OCH_2_CH_3_)_9_TRIOL^Py^]^1–^ equivalents. (F) Steady-state (blue) and time-resolved (red) PL quenching. The blue and red dotted lines are a guide to the eye. τ_0_ is the amplitude-weighted average PL lifetime of just the QDs + MS, and τ is the amplitude-weighted average lifetime with added [V_6_O_7_(OCH_2_CH_3_)_9_TRIOL^Py^]^1–^. The lifetimes in (C,F) are extracted from separate triple exponential tail fits independent of our analysis with the Tachiya model. Short time scale TCSPC traces are shown in Figure S5.

With the important controls performed, we turned our attention to the investigation of HT to [V_6_O_7_(OCH_2_CH_3_)_9_TRIOL^Py^]^1–^ in an attempt to understand the impact of the installation of a pyridine functional group on the efficiency of charge transfer in these systems. As such, we evaluated the addition of increasing equivalents of [V_6_O_7_(OCH_2_CH_3_)_9_TRIOL^Py^]^1–^ to OA-CdSe QDs treated with 20 equiv of MS under analogous conditions to those described above. Stern–Volmer analysis of steady-state PL quenching data reveals that there is a nonlinear dependence of PL quenching on cluster concentration. At low equivalents of [V_6_O_7_(OCH_2_CH_3_)_9_TRIOL^Py^]^1–^ (e.g., 1–5 equiv), PL quenching of the QD by the POV-alkoxide is stronger than that observed for either [V_6_O_7_(OCH_2_CH_3_)_12_]^1–^ and [V_6_O_7_(OCH_2_CH_3_)_9_TRIOL^Me^]^1–^ (Figure 4D,F). This observation suggests that the pyridine functional group appended to the surface of the POV-alkoxide increases the number of cluster-QD associations, particularly at low concentrations of the redox mediator. However, at higher concentrations of [V_6_O_7_(OCH_2_CH_3_)_9_TRIOL^Py^]^1–^ (e.g., 10–20 equiv), PL quenching appears to plateau, reaching a maximum HT efficiency of ∼53%. This observation indicates saturation behavior at these surface accessible sites on the QD. The functional group influences how efficiently the association occurs, but it does not overcome the steric hindrance imposed in the system from the ethoxide ligand on the POV-alkoxide cluster. We note that a control experiment in which 4-methylpyridine was added to OA-CdSe treated with MS results in no measurable PL quenching (Figure S4). This result indicates that pyridine alone does not facilitate hole transfer. Enhanced quenching is only observed when the pyridine functional group is attached to the redox-active POV-alkoxide.

The enhanced quenching observed at lower equivalents of [V_6_O_7_(OCH_2_CH_3_)_9_TRIOL^Py^]^1–^ suggests that the installation of the pyridine functional group promotes stronger association of the cluster to the QD surface. This observation is consistent with the fact that prior studies have established that pyridine can directly coordinate to surface Cd^2+^ sites in CdSe nanocrystals. For example, early work by Murray and co-workers demonstrated that the exposure of TOPO-capped CdSe QDs in the solvent pyridine results in ligand exchange with pyridine, allowing the nanocrystals to be soluble in polar organic solvents.^26^ Another study by Dzhagn and co-workers confirmed that pyridine treatment replaces natively capped OA ligand on CdSe QDs, as shown by ^1^H NMR spectroscopy.^60^ In light of this work, we propose that the incorporation of the TRIOL^Py^ ligand at the surface of the POV-alkoxide provides a pathway for enhanced association with the CdSe QD surface by coordination to surface-exposed Cd^2+^ sites.

To study the excited-state dynamics of hole transfer to the TRIOL-capped POV-alkoxides, time-resolved PL measurements were performed. In the case of [V_6_O_7_(OCH_2_CH_3_)_9_TRIOL^Me^]^1–^, PL lifetimes remain mostly unchanged upon addition of further equivalents of POV-alkoxide (Figure 4B). This is observed despite the noticeable increase in steady-state PL quenching. Notably, these results are nearly identical to those observed for the POV-ethoxide, [V_6_O_7_(OCH_2_CH_3_)_12_]^1–^ (Figure 3B), confirming that the reduction in the symmetry of the vanadium oxide assembly does not influence charge transfer kinetics from CdSe QDs.

In contrast, the time-resolved PL experiments indicate that the lifetimes of the excited-state of the CdSe QDs change in the presence of [V_6_O_7_(OCH_2_CH_3_)_9_TRIOL^Py^]^1–^ (Figure 4E). Indeed, upon the addition of increased equivalents of POV-alkoxide, slight shortening of the PL lifetimes is observed (Table S4). This result stands in contrast to time-resolved photoluminescence decay kinetics measured for both [V_6_O_7_(OCH_2_CH_3_)_12_]^1–^ and [V_6_O_7_(OCH_2_CH_3_)_9_(TRIOL^Me^)]^1–^, where no observable change in the normalized PL kinetics is observed (Figures 3E and 4C, respectively). We note, however, that comparison of the Stern-Volmer plots obtained for all three POV-alkoxides indicates that quenching is dominated by strongly associated QD-cluster adducts, as opposed to purely dynamic collisions.^65^

Comparison of steady-state and time-resolved Stern–Volmer data reveals differences in the extent of the QD-cluster associations between the two distinct TRIOL-capped POV-alkoxides. In the case of [V_6_O_7_(OCH_2_CH_3_)_9_(TRIOL^Me^)]^1–^, the PL lifetime changes only slightly with added cluster, even in the presence of significant PL quenching in the steady state. This data indicates that a large fraction of the QDs form strongly associated complexes with the POV-alkoxide that have an exceptionally fast hole transfer rate. With respect to interactions between diffusing clusters and QDs, on average only 0.13 ± 0.02 POV-alkoxides associate with the surface of the nanocrystal (Figure 5A and Table S3). When a similar analysis was performed for the quenching behaviors of [V_6_O_7_(OCH_2_CH_3_)_9_(TRIOL^Py^)]^1–^, we note that the maximum number of QD-cluster associations increases to 0.19 ± 0.06 (we note that the maximum number of acceptors is observed following addition of 10 equiv of POV-alkoxide, though within error Table S4). The increased number of acceptors observed from 1 to 5 equiv in the case of the pyridine-capped POV-alkoxide reflects a modest increase in the QD-cluster associations observed in steady-state. While this increase is small, it is significant and indicates that the pyridine-backed assembly has a stronger association with the surface of the CdSe QD under identical reaction conditions.

**Figure 5:**
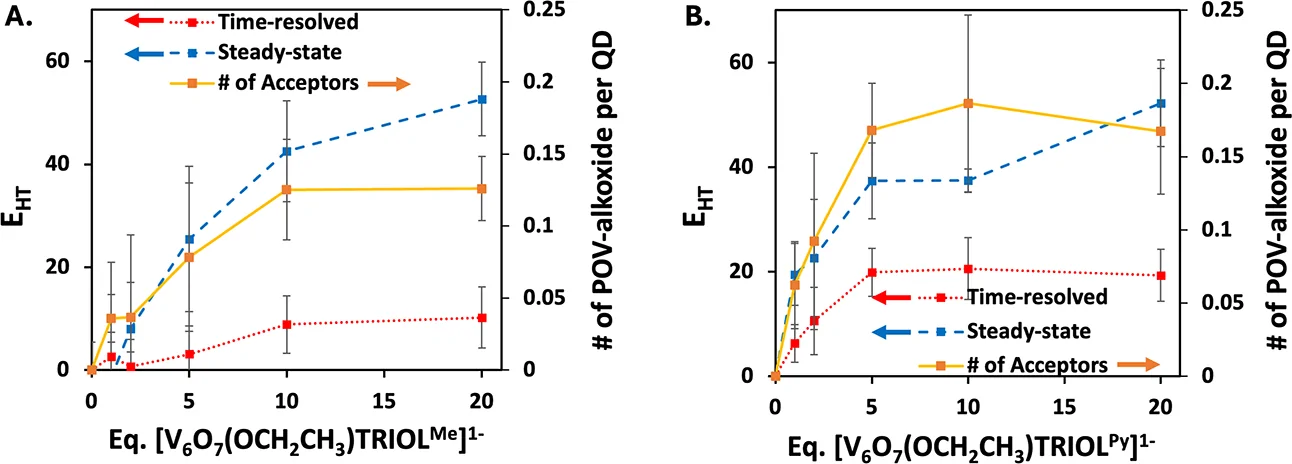
(A) PL quenching efficiency of OA-CdSe QDs +20 eq of Meerwein’s salt with an increasing number of [V_6_O_7_(OCH_2_CH_3_)_9_(TRIOL^Me^)]^1–^ equivalents based on steady-state E_HT_ (quenching) (blue dashed line) and time-resolved E_HT_ (lifetimes) (red dotted line) measurements with the number of acceptors (yellow line) and (B) (A) PL quenching efficiency of OA-CdSe QDs +20 eq of Meerwein’s salt with an increasing number of [V_6_O_7_(OCH_2_CH_3_)_9_(TRIOL^Py^)]^1–^ equivalents based on steady-state E_HT_ (quenching) (blue dashed line) and time-resolved E_HT_ (lifetimes) (red dotted line) measurements with the number of acceptors (yellow line) The number of acceptors are determined from TRPL measurements as described.

While the maximum number of QD-acceptor contacts differs between the methyl- (0.13 ± 0.02) and pyridine- (0.19 ± 0.06) backed POV-alkoxides, analysis of the kinetic fitting parameters of the time-resolved data (see Tables S3 and S4 in Supporting Information) indicates that hole transfer kinetics (**τ**_**1HT**_) for [V_6_O_7_(OCH_2_CH_3_)_9_(TRIOL^Py^)]^1–^ (0.71 ± 0.18 ns) is significantly longer than that of [V_6_O_7_(OCH_2_CH_3_)_9_(TRIOL^Me^)]^1–^ (0.09 ± 0.05 ns). This result is surprising, as we anticipated that an increase in cluster association at the nanocrystal surface would track linearly with the increase in kinetics of charge transfer. To explain this observation, we considered the types of interactions of the two TRIOL-substituted POV-alkoxides with the nanocrystal surface. Evaluation of the time-resolved PL decay kinetics reveals a charge transfer mechanism faster than the IRF is operative for [V_6_O_7_(OCH_2_CH_3_)_9_(TRIOL^Me^)]^1–^. In contrast, the pyridine-backed assembly exhibits some changes in the overall traces that would be consistent with a small component of diffusive interactions with the nanocrystal surface. As discussed above in the comparative analysis of the POV-methoxide and -ethoxide, the presence of these dynamic-like charge-transfer mechanisms appears to inhibit hole-transfer kinetics in these systems.

It is important to recall that the nanocrystals investigated have been treated with 20 equiv of MS, removing a subset of ligands from the surface of the QD. When these ligands are removed as methyl oleate, the positive charge left at cadmium ions at the surface of the QD is compensated by noncoordinating BF_4_^1–^ ions. We note that all POV-alkoxides studied in this work are negatively charged, and thus are capable of displacing the BF_4_^1–^ ions from the surface of the nanocrystal. This ionic contact has been described in our prior work, and is credited with the efficient charge transfer observed from QD to POV-alkoxide. In the case of the POV-alkoxides studied here that lack a molecular anchor to promote specific association with the surface of the nanocrystal, these interactions are random. This observation is consistent with similar **τ**_**1HT**_ values obtained for both [V_6_O_7_(OCH_2_CH_3_)_12_]^1–^ (0.07 ± 0.02 ns, Table S1) and [V_6_O_7_(OCH_2_CH_3_)_9_(TRIOL^Me^)]^1–^. However, in the case of [V_6_O_7_(OCH_2_CH_3_)_9_(TRIOL^Py^)]^1–^, cluster attachment is specific; the pyridine functional group directs association of the vanadium oxide assembly through the TRIOL ligand, increasing the distance of the hole acceptor from the nanocrystal surface. Thus, while pyridine functionalization leads to a slightly higher number of QD-cluster contacts, the observed hole transfer kinetics indicate that this specific interaction inhibits the rates of hole transfer, likely due to an increase in distance (and electronic coupling) of the vanadium oxide core from the CdSe QD.

## Conclusion

In this work, we demonstrate that the surface ligand environment of POV-alkoxide clusters provides a strategy to modulate interfacial hole transfer from CdSe QDs. By using controlled MS stripping treatment to partially removal native oleate ligands, we were able to hold the QD surface accessibility constant, allowing direct evaluation of how changes to the molecular acceptor influence hole transfer efficiency. Our results establish that the steric environment at the cluster surface governs the probability of QD-cluster association, while the incorporation of a functional anchoring group can further alter how the acceptor interacts with the nanocrystal surface.

Comparison across the POV-alkoxide series reveals that modifications to cluster surface ligands directly influence QD-cluster association. The most substantial impact is observed when the bridging alkoxide ligands are lengthened; [V_6_O_7_(OCH_3_)_12_]^1–^ quenches the PL of the CdSe QD most efficiently. In the case of these experiments, increasing the alkoxide chain length from -methoxide to -ethoxide results in a decrease in the total steady-state PL quenching observed and a lower number of associated QD-cluster contacts, as quantified by Poissonian analysis of time-resolved measurements. These findings indicate that the decrease in hole transfer efficiency of the all-ethoxide cluster is due to reduced interfacial association from steric hindrance rather than changes in intrinsic cluster electronics.

Installation of capping ligands at the surface of the POV-alkoxide provides an additional handle for modulating cluster-QD interactions. While [V_6_O_7_(OCH_2_CH_3_)_9_(TRIOL^Me^)]^1–^ exhibits similar behavior to POV-ethoxide, [V_6_O_7_(OCH_2_CH_3_)_9_(TRIOL^Py^)]^1–^ shows enhancement in quenching efficiency at lower equivalents of cluster as a result of increased numbers of strongly associated QD-cluster pairs. However, analysis of the intrinsic hole transfer kinetics from photoexcited CdSe QDs to [V_6_O_7_(OCH_2_CH_3_)_9_(TRIOL^Py^)]^1–^ shows slower hole transfer once associated with the QD. We hypothesize that this is a result of the pyridine group dictating how the cluster is positioned relative to the QD surface, which influences electronic coupling between the pair. Taken together, these results demonstrate that both the extent of interfacial association and arrangement at the interface contribute to the observed hole transfer behavior.

## Supplementary Material



## Notes

Additional analysis for steady-state and time-resolved photoluminescence, Stern–Volmer fitting, and hole transfer efficiency calculations. Supporting figures and tables summarizing TCSPC fitting parameters and extracted kinetic values are also provided (PDF) (PDF)

## References

[1] Wu K. F., Lian T. Q. (2016). Quantum confined colloidal nanorod heterostructures
for solar-to-fuel conversion. Chem. Soc. Rev..

[2] Nozik A. J. (2002). Quantum
dot solar cells. Physica E.

[3] Kamat P. V. (2012). Boosting
the Efficiency of Quantum Dot Sensitized Solar Cells through Modulation
of Interfacial Charge Transfer. Acc. Chem. Res..

[4] Santra P. K., Kamat P. V. (2013). Tandem-Layered Quantum Dot Solar Cells: Tuning the
Photovoltaic Response with Luminescent Ternary Cadmium Chalcogenides. J. Am. Chem. Soc..

[5] Spittel D., Poppe J., Meerbach C., Ziegler C., Hickey S. G., Eychmüller A. (2017). Absolute Energy
Level Positions in CdSe Nanostructures
from Potential-Modulated Absorption Spectroscopy (EMAS). ACS Nano.

[6] Leatherdale C. A., Woo W. K., Mikulec F. V., Bawendi M. G. (2002). On the absorption
cross section of CdSe nanocrystal quantum dots. J. Phys. Chem. B.

[7] Schimpf A. M., Knowles K. E., Carroll G. M., Gamelin D. R. (2015). Electronic Doping
and Redox-Potential Tuning in Colloidal Semiconductor Nanocrystals. Acc. Chem. Res..

[8] Burke R., Cogan N. M. B., Oi A., Krauss T. D. (2018). Recovery of Active
and Efficient Photocatalytic H_2_ Production for CdSe Quantum
Dots. J. Phys. Chem. C.

[9] Burke R., Bren K. L., Krauss T. D. (2021). Semiconductor nanocrystal
photocatalysis
for the production of solar fuels. J. Chem.
Phys..

[10] Darwent J. R., Porter G. (1981). Photochemical Hydrogen-Production Using Cadmium-Sulfide
Suspensions in Aerated Water. J. Chem. Soc.
Chem. Commun..

[11] Yan H. J., Yang J. H., Ma G. J., Wu G. P., Zong X., Lei Z. B., Shi J. Y., Li C. (2009). Visible-light-driven
hydrogen production with extremely high quantum efficiency on Pt-PdS/CdS
photocatalyst. J. Catal..

[12] Shaikh A. F., Arbuj S. S., Tamboli M. S., Naik S. D., Rane S. B., Kale B. B. (2017). ZnSe/ZnO Nano-Heterostructures for
Enhanced Solar Light
Hydrogen Generation. ChemistrySelect.

[13] Wang K. H., Wang C., Tao Y., Tang Z. K., Benetti D., Vidal F., Liu Y., Rummeli M. H., Zhao H. G., Rosei F., Sun X. (2024). High-performance Photoelectrochemical
Hydrogen Production Using Asymmetric Quantum Dots. Adv. Funct. Mater..

[14] Zhu S. C., Xiao F. X. (2023). Transition Metal
Chalcogenides Quantum Dots: Emerging
Building Blocks toward Solar-to-Hydrogen Conversion. ACS Catal..

[15] Zhong H. Z., Bai Z. L., Zou B. S. (2012). Tuning
the Luminescence Properties
of Colloidal I-III-VI Semiconductor Nanocrystals for Optoelectronics
and Biotechnology Applications. J. Phys. Chem.
Lett..

[16] Li S. J., Shao H. Y., Ding K., Yao M. J., Dou M. H., Cheng Y. Y., Zhang K. Y., Yang N. X., Ma Y. Z., Chen Y. Y. (2023). Regulating Deep
and Shallow Defects of CuInS Quantum
Dots To Enhance Photocatalytic Hydrogen Production. ACS Appl. Nano Mater..

[17] Acharya K. P., Zamkov M., Huang J. M. (2026). Advances in Colloidal InP-Based Quantum
Dots for Photocatalytic Hydrogen Evolution. ACS Omega.

[18] Yu S., Fan X. B., Wang X., Li J. G., Zhang Q., Xia A. D., Wei S. Q., Wu L. Z., Zhou Y., Patzke G. R. (2018). Efficient photocatalytic
hydrogen evolution with ligand
engineered all-inorganic InP and InP/ZnS colloidal quantum dots. Nat. Commun..

[19] Gueret R., Poulard L., Oshinowo M., Chauvin J., Dahmane M., Dupeyre G., Lainé P. P., Fortage J., Collomb M. N. (2018). Challenging
the [Ru­(bpy)]^2+^ Photosensitizer with a Triazatriangulenium
Robust Organic Dye for Visible-Light-Driven Hydrogen Production in
Water. ACS Catal..

[20] Lynch P. G., Richards H., Wustholz K. L. (2019). Unraveling the Excited-State
Dynamics
of Eosin Y Photosensitizers Using Single-Molecule Spectroscopy. J. Phys. Chem. A.

[21] Peng J. B., Qi X. X., Wu X. F. (2016). Visible Light-Induced Carbonylation
Reactions with Organic Dyes as the Photosensitizers. ChemSusChem.

[22] Gust D., Moore T. A., Moore A. L. (2001). Mimicking
photosynthetic solar energy
transduction. Acc. Chem. Res..

[23] Nozik A. J. (2001). Spectroscopy
and hot electron relaxation dynamics in semiconductor quantum wells
and quantum dots. Annu. Rev. Phys. Chem..

[24] Yu W. W., Qu L. H., Guo W. Z., Peng X. G. (2004). Experimental determination
of the extinction coefficient of CdTe, CdSe and CdS nanocrystals. Chem. Mater..

[25] Owen J. S., Chan E. M., Liu H. T., Alivisatos A. P. (2010). Precursor
Conversion Kinetics and the Nucleation of Cadmium Selenide Nanocrystals. J. Am. Chem. Soc..

[26] Murray C.
B., Norris D. J., Bawendi M. G. (1993). Synthesis and Characterization of
Nearly Monodisperse Cde (E = S, Se, Te) Semiconductor Nanocrystallites. J. Am. Chem. Soc..

[27] Edwards E. H., Fertig A. A., McClelland K. P., Meidenbauer M. T., Chakraborty S., Krauss T. D., Bren K. L., Matson E. M. (2020). Enhancing
the activity of photocatalytic hydrogen evolution from CdSe quantum
dots with a polyoxovanadate cluster. Chem. Commun..

[28] Burke R., Chakraborty S., McClelland K. P., Jelusic J., Matson E. M., Bren K. L., Krauss T. D. (2021). Light-driven hydrogen production
with CdSe quantum dots and a cobalt glutathione catalyst. Chem. Commun..

[29] Han Z. J., Qiu F., Eisenberg R., Holland P. L., Krauss T. D. (2012). Robust Photogeneration
of H_2_ in Water Using Semiconductor Nanocrystals and a Nickel
Catalyst. Science.

[30] Xiang X. L., Wang L. X., Zhang J. J., Cheng B., Yu J. G., Macyk W. (2022). Cadmium Chalcogenide (CdS, CdSe, CdTe) Quantum Dots for Solar-to-Fuel
Conversion. Adv. Photon. Res..

[31] Liu J., Ren Y. Y., Wu J., Xia W., Deng B. Y., Wang F. (2021). Hybrid artificial photosynthetic systems constructed using quantum
dots and molecular catalysts for solar fuel production: development
and advances. J. Mater. Chem. A.

[32] Kamat P. V., Christians J. A., Radich J. G. (2014). Quantum Dot Solar Cells: Hole Transfer
as a Limiting Factor in Boosting the Photoconversion Efficiency. Langmuir.

[33] Zhu H. M., Yang Y., Wu K. F., Lian T. Q. (2016). Charge Transfer
Dynamics from Photoexcited Semiconductor Quantum Dots. Annu. Rev. Phys. Chem..

[34] Gómez-Campos F.
M., Califano M. (2012). Hole Surface
Trapping in CdSe Nanocrystals: Dynamics,
Rate Fluctuations, and Implications for Blinking. Nano Lett..

[35] Kodaimati M. S., McClelland K. P., He C., Lian S. C., Jiang Y. S., Zhang Z. Y., Weiss E. A. (2018). Viewpoint:
Challenges in Colloidal
Photocatalysis and Some Strategies for Addressing Them. Inorg. Chem..

[36] McClelland K. P., Weiss E. A. (2019). Selective Photocatalytic
Oxidation of Benzyl Alcohol
to Benzaldehyde or C-C Coupled Products by Visible-Light-Absorbing
Quantum Dots. ACS App. Energy Mater..

[37] Zhu H. M., Song N. H., Lian T. Q. (2013). Charging of Quantum
Dots by Sulfide
Redox Electrolytes Reduces Electron Injection Efficiency in Quantum
Dot Sensitized Solar Cells. J. Am. Chem. Soc..

[38] Boulesbaa A., Issac A., Stockwell D., Huang Z., Huang J., Guo J., Lian T. (2007). Ultrafast charge separation at CdS quantum dot/rhodamine
B molecule interface. J. Am. Chem. Soc..

[39] Jasieniak J., Califano M., Watkins S. E. (2011). Size-Dependent Valence and Conduction
Band-Edge Energies of Semiconductor Nanocrystals. ACS Nano.

[40] Klimov V. I. (2000). Optical
nonlinearities and ultrafast carrier dynamics in semiconductor nanocrystals. J. Phys. Chem. B.

[41] Lian S. C., Weinberg D. J., Harris R. D., Kodaimati M. S., Weiss E. A. (2016). Subpicosecond Photoinduced Hole Transfer
from a CdS
Quantum Dot to a Molecular Acceptor Bound Through an Exciton-Delocalizing
Ligand. ACS Nano.

[42] Click S. M., Jilek M. R., Bird O. F., Prugh N., Flinkingshelt F. A., Lamb Y. M. L., Yang J. Y., Miller K. A., Dukovic G. (2026). Exceptionally
long-lived charge-separated states in CdS nanocrystals with a covalently
bound phenothiazine derivative. Chem.

[43] Yan C., Weinberg D., Jasrasaria D., Kolaczkowski M. A., Liu Z. J., Philbin J. P., Balan A. D., Liu Y., Schwartzberg A. M., Rabani E., Alivisatos A. P. (2021). Uncovering
the Role of Hole Traps in Promoting Hole Transfer from Multiexcitonic
Quantum Dots to Molecular Acceptors. ACS Nano.

[44] Pearce O. M., Duncan J. S., Damrauer N. H., Dukovic G. (2018). Ultrafast
Hole Transfer
from CdS Quantum Dots to a Water Oxidation Catalyst. J. Phys. Chem. C.

[45] Zang H. D., Routh P. K., Alam R., Maye M. M., Cotlet M. (2014). Core size
dependent hole transfer from a photoexcited CdSe/ZnS quantum dot to
a conductive polymer. Chem. Commun..

[46] Song N. H., Zhu H. M., Jin S. Y., Lian T. Q. (2011). Hole Transfer from
Single Quantum Dots. ACS Nano.

[47] Olshansky J. H., Ding T. X., Lee Y. V., Leone S. R., Alivisatos A. P. (2015). Hole Transfer
from Photoexcited Quantum Dots: The Relationship between Driving Force
and Rate. J. Am. Chem. Soc..

[48] Ding T. N. X., Olshansky J. H., Leone S. R., Alivisatos A. P. (2015). Efficiency
of Hole Transfer from Photoexcited Quantum Dots to Covalently Linked
Molecular Species. J. Am. Chem. Soc..

[49] Huang J. E., Huang Z. Q., Jin S. Y., Lian T. Q. (2008). Exciton Dissociation
in CdSe Quantum Dots by Hole Transfer to Phenothiazine. J. Phys. Chem. C.

[50] Dutta P., Beaulac R. (2016). Photoluminescence Quenching
of Colloidal CdSe and CdTe
Quantum Dots by Nitroxide Free Radicals. Chem.
Mater..

[51] Dutta P., Tang Y. H., Mi C. J., Saniepay M., McGuire J. A., Beaulac R. (2019). Ultrafast hole extraction
from photoexcited colloidal
CdSe quantum dots coupled to nitroxide free radicals. J. Chem. Phys..

[52] Cogan N. M. B., McClelland K. P., Peter C. Y. M., Carmenate
Rodríguez C., Fertig A. A., Amin M., Brennessel W. W., Krauss T. D., Matson E. M. (2023). Efficient Hole Transfer from CdSe
Quantum Dots Enabled by Oxygen-Deficient Polyoxovanadate-Alkoxide
Clusters. Nano Lett..

[53] Peter C. Y. M., Carmenate Rodríguez C., Gorski H. N., Phinney E. O., Krauss T. D., Matson E. M. (2025). Modulating Hole
Transfer from CdSe
Quantum Dots by Manipulating the Surface Ligand Density. Nano Lett..

[54] Schurr B. E., Nachtigall O., VanGelder L. E., Drappeau J., Brennessel W. W., Matson E. M. (2019). Consequences of ligand derivatization on the electronic
properties of polyoxovanadate-alkoxide clusters. J. Coord. Chem..

[55] VanGelder L. E., Cook T. R., Matson E. M. (2019). Progress
in the Design of Polyoxovanadate-Alkoxides
as Charge Carriers for Nonaqueous Redox Flow Batteries. Comments Inorg. Chem..

[56] VanGelder L. E., Petel B. E., Nachtigall O., Martinez G., Brennessel W. W., Matson E. M. (2018). Organic Functionalization
of Polyoxovanadate-Alkoxide
Clusters: Improving the Solubility of Multimetallic Charge Carriers
for Nonaqueous Redox Flow Batteries. ChemSusChem.

[57] Chen Q., Goshorn D. P., Scholes C. P., Tan X. L., Zubieta J. (1992). Coordination
Compounds of Polyoxovanadates with a Hexametalate Core - Chemical
and Structural Characterization of [V­(V)_6_O_13_((OCH_2_)_3_CR)_2_]^2‑^, [V­(V)_6_O_11_(OH)_2_((OCH_2_)_3_CR)_2_], [V­(IV)_4_V­(V)_2_O_9_(OH)_4_((OCH_2_)_3_CR)_2_]^2‑^, and [V­(IV)_6_O_7_(OH)_6_((OCH_2_)_3_CR)_2_]^2‑^. J. Am. Chem. Soc..

[58] Qin C., Zubieta J. (1993). A Novel Hexavanadate Core-Synthesis and Structure of
the Mixed-Valence Cluster [V_6_O_8_((OCH_2_)_3_CEt)_2_((OCH_2_)_2_C­(CH_2_OH)­(Et))_4_]^2‑^ and a Comparison
with the Hexametallate Core of [V_6_O_13_(OMe)_3_((OCH_2_)_3_CCH_2_OH)]^2‑^. J. Chem. Soc. Chem. Comm..

[59] Lokteva I., Radychev N., Witt F., Borchert H., Parisi J., Kolny-Olesiak J. (2010). Surface Treatment of CdSe Nanoparticles for Application
in Hybrid Solar Cells: The Effect of Multiple Ligand Exchange with
Pyridine. J. Phys. Chem. C.

[60] Dzhagan V. M., Lokteva I., Himcinschi C., Kolny-Olesiak J., Valakh M. Y., Schulze S., Zahn D. R. T. (2011). The influence
of pyridine ligand onto the structure and phonon spectra of CdSe nanocrystals. J. Appl. Phys..

[61] Bartolo M. D., Brisbin R. P., Fettinger J. C., Ghosh S., Baxter R. D. (2020). Impact
of Bis­(imino)­pyridine Ligands on Mesoscale Properties of CdSe/ZnS
Quantum Dots. J. Phys. Chem. C.

[62] Peter C. Y. M., Schreiber E., Proe K. R., Matson E. M. (2023). Surface
ligand length
influences kinetics of H-atom uptake in polyoxovanadate-alkoxide clusters. Dalton Trans..

[63] Knauf R. R., Lennox J. C., Dempsey J. L. (2016). Quantifying
Ligand Exchange Reactions
at CdSe Nanocrystal Surfaces. Chem. Mater..

[64] Dang V. Q., Teets T. S. (2025). A practical guide
to measuring and reporting photophysical
data. Dalton T.

[65] Lakowicz, J. R. Quenching of Fluorescence. In Principles of Fluorescence Spectroscopy; Springer US, 1983; pp 257–301.

[66] Sadhu S., Tachiya M., Patra A. (2010). A Stochastic
Model for Energy Transfer
from CdS Quantum Dots/Rods (Donors) to Nile Red Dye (Acceptors). J. Phys. Chem. C.

[67] Petel B. E., Meyer R. L., Brennessel W. W., Matson E. M. (2019). Oxygen atom transfer
with organofunctionalized polyoxovanadium clusters: O-atom vacancy
formation with tertiary phosphanes and deoxygenation of styrene oxide. Chem. Sci..

[68] Schreiber E., Hartley N. A., Brennessel W. W., Cook T. R., McKone J. R., Matson E. M. (2019). An Organofunctionalized
Polyoxovanadium Cluster as
a Molecular Model of Interfacial Pseudocapacitance. ACS Appl. Energy Mater..

[69] Spandl J., Daniel C., Brüdgam I., Hartl H. (2003). Synthesis and structural
characterization of redox-active dodecamethoxoheptaoxohexavanadium
clusters. Angew. Chem., Int. Ed..

[70] Rosen E. L., Buonsanti R., Llordes A., Sawvel A. M., Milliron D. J., Helms B. A. (2012). Exceptionally Mild Reactive Stripping
of Native Ligands
from Nanocrystal Surfaces by Using Meerwein’s Salt. Angew. Chem., Int. Ed..

[71] Chang C., Orchard K., Martindale B., Reisner E. (2016). Ligand removal from
CdS quantum dots for enhanced photocatalytic H_2_ generation
in pH neutral water. J. Mater. Chem. A.

[72] Duong J. T., Bailey M. J., Pick T. E., McBride P. M., Rosen E. L., Buonsanti R., Milliron D. J., Helms B. A. (2012). Efficient polymer
passivation of ligand-stripped nanocrystal surfaces. J. Polymer Sci. Pol. Chem..

[73] Pelcher K. E., Milleville C. C., Wangoh L., Chauhan S., Crawley M. R., Marley P. M., Piper L. F. J., Watson D. F., Banerjee S. (2015). Integrating
β-PbV_2_O_5_ Nanowires with CdSe Quantum Dots:
Toward Nanoscale Heterostructures with Tunable Interfacial Energetic
Offsets for Charge Transfer. Chem. Mater..

