# Supplementary material for: Surface Ligand Composition of Polyoxovanadate-Alkoxides Influences Hole Transfer from CdSe Quantum Dots
Source: Inorg Chem. 2026 Oct 15;65(42):24660–71. doi: 10.1021/acs.inorgchem.6c01486 (PMC13715574; doi:10.1021/acs.inorgchem.6c01486)
Supplement: Supplementary file 1 [file ic6c01486_si_001.pdf]

Electronic Supplementary Information for

Surface Ligand Composition of Polyoxovanadate-alkoxides Influences Hole Transfer from CdSe Quantum Dots

Chari Y. M Peter[a‡], Benjamin Fedrigo[b‡], John J. Caruso[a], Nicholas B. Flemings[a], Margaret C. Hintz[a], Todd D. Krauss[a,b]* and Ellen M. Matson[a]*

[a] Department of Chemistry, University of Rochester, Rochester NY 14627, USA.

[b] Institute of Optics, University of Rochester, Rochester, NY 14627, USA.

[‡] These authors contributed equally to this work.

Table of Contents:

Experimental Methods: 3

General Considerations 3

OA-CdSe QDs Synthesis 3

Synthesis of $[V_6O_7(OCH_3)_{12}]^{1-}$ 3

Synthesis of $[V_6O_7(OCH_2CH_3)_{12}]^{1-}$ 3

Synthesis of $[V_6O_7(OCH_2CH_3)_9TRIOL^{Me}]^{1-}$ 3

Synthesis of $[V_6O_7(OCH_2CH_3)_9TRIOL^{Py}]^{1-}$ 3

Time-Correlated Single Photon Counting 4

Photoluminescence Quenching 4

Stern-Volmer Quenching Constant[4] 4

Hole transfer efficiency calculations 5

PL decay dynamics analysis 5

Number of molecular acceptors estimation 5

Supporting Figures

Figure S1. Normalized PL intensity of $[V_6O_7(OCH_2CH_3)_{12}]^{1-}$ + OA-CdSe 7

Figure S2. Normalized PL intensity of $[V_6O_7(OCH_2CH_3)_9TRIOL^{Me}]^{1-}$ + OA-CdSe 7

Figure S3. Normalized PL intensity of $[V_6O_7(OCH_2CH_3)_9TRIOL^{Py}]^{1-}$ + OA-CdSe 8

Figure S4. Normalized PL intensity of free 4-methyl pyridine +10 eq MS treated OA-CdSe 8

Figure S 5 Short timescale TCSPC traces for OA-CdSe QDs + 20 eq MS + (**A**) $[V_6O_7(OCH_2CH_3)_{12}]^{1-}$, (**B**) $[V_6O_7(OCH_3)_{12}]^{1-}$, (**C**) $[V_6O_7(OCH_2CH_3)_9TRIOL^{Me}]^{1-}$, and (**D**) $[V_6O_7(OCH_2CH_3)_9TRIOL^{Py}]^{1-}$ 9

Figure S6. ^{1}H NMR spectra of OA-CdSe QDs with 0-20 eq MS in toluene-d_8. 10

Figure S7. Comparison of HT efficiencies for OA-CdSe QDs + 20eq MS + (**A**) $V_6O_7(OCH_2CH_3)_{12}{}^{1-}$ and (**B**) $V_6O_7(OCH_3)_{12}{}^{1-}$ 10

Figure S8. Instrument response function for TCSPC measurements. 11

Figure S9. Example of untruncated TCSPC curve for OA-CdSe QDs + 20 eq MS.. 11

Supporting Tables

Table S1. Summary of TCSPC Fitting Parameters for OA-CdSe QDs + 20 eq MS + $[V_6O_7(OCH_2CH_3)_{12}]^{1-}$ ..... **Error! Bookmark not defined.**

Table S2. Summary of TCSPC Fitting Parameters for OA-CdSe QDs + 20 eq MS + $[V_6O_7(OCH_3)_{12}]^{1-}$ 12

Table S3. Summary of TCSPC Fitting Parameters for OA-CdSe QDs + 20eq MS + $[V_6O_7(OCH_2CH_3)_9TRIOL^{Me}]^{1-}$ 14

Table S4. Summary of TCSPC Fitting Parameters for OA-CdSe QDs + 20eq MS + $[V_6O_7(OCH_2CH_3)_9TRIOL^{Py}]^{1-}$ 15

Table S5. Quantified bound, free methyl oleate, and total ligand per QD via ^{1}H NMR spectroscopy 16

Experimental Methods:

General Considerations

Chemicals were purchased and used without further purification except where noted. Cadmium (II) oxide (CdO, 99.99%), selenium (Se, 100 mesh, 99.99%), 1-octadecene (ODE, 90%), oleic acid (OA, 90%), trimethyl oxonium tetrafluoroborate (MS, 95%), and tetrabutylammonium borohydride ($TBABH_4$, 98%), were purchased from Sigma-Aldrich and used as received. Methylene Chloride (DCM) (Stabilized/Certified ACS, Fisher Chemical™) was purchased from Fischer scientific. Tetrabutylammonium Hexafluorophosphate (TBA PF6, 98%) was purchased from Sigma-Aldrich, recrystallized thrice using hot methanol, and stored under dynamic vacuum for a minimum of two days prior to use.

OA-CdSe QDs Synthesis

To a dry, three-neck round-bottom flask, 3mL of OA, 10 mL of ODE, and 0.514 g of CdO (0.514 g, 4.00 mmol) were added. The flask was placed under an N_2 atmosphere and heated to 260°C while stirring at 1100 rpm under reflux. While the solution was heating, the Se precursor was prepared by sonication of 0.68 g of Se in 10 mL ODE until a uniform suspension was formed. Once the Cd precursor became clear and the temperature reached 260°C, 1 mL Se precursor was rapidly injected. The QDs were allowed to grow for 20 seconds at 250 °C before thermal quenching with a heat gun (on the cool setting) and water bath once the temperature reached below 180 °C. In order to remove the unused precursor, the solution was washed with ethanol in a 6:1 (v/v) ratio to QDs followed by centrifugation at 5000 rpm for 15 minutes. The clear supernatant was then discarded, and the pellet was redispersed in minimal hexanes. We repeated the washing once more and stored the final QD solution in 15 mL Hexanes.

Synthesis of $[V_6O_7(OCH_3)_{12}]^{1-}$

Cluster was prepared according to previous literature.[1]

Synthesis of $[V_6O_7(OCH_2CH_3)_{12}]^{1-}$

Cluster was prepared according to previous literature.[2]

Synthesis of $[V_6O_7(OCH_2CH_3)_9TRIOL^{Me}]^{1-}$

Cluster was prepared according to previous literature.[2]

Synthesis of $[V_6O_7(OCH_2CH_3)_9TRIOL^{Py}]^{1-}$

Cluster was prepared according to previous literature.[2]

Time-Correlated Single Photon Counting

Ensemble TCSPC measurements were acquired using a home-built optical setup featuring an inverted microscope (Nikon, TE-200U) with a 10x air objective (Nikon, NA 0.3) and a 488 nm dichroic mirror (Semrock, Inc.). Samples, which had been transferred to quartz cuvettes with 1 mm thick windows, were photoexcited by a defocused laser beam provided by a pulsed (5 MHz) supercontinuum source (FYLA Iceblink), with 488 nm excitation selected through bandpass and shortpass filters. Power-dependent studies were performed to ensure that excitation remained in the single-exciton regime and the same excitation power used for all samples. Photoluminescence (PL) emission was collected by a single photon avalanche diode detector (Micro Photon Devices, PDM Series, 50 µm active sensing area diameter) with a fast-timing resolution (56 ps). All components of the TCSPC setup were incorporated in the PicoHarp 300 module (PicoQuant). The instrument response function was collected from residual scattering of the light from the pulsed laser diode. To collect lifetime decay curves, 488 nm longpass filters were placed in the emission pathway to block scattered excitation light. Data collection was halted once a 1000 count maximum was reached and the count rate was kept below 1 % the laser repetition rate. Decay curves corresponding to OA-CdSe QDs + n eq MS without the quencher were first fit to a triple exponential decay to establish the dynamics before PL quenching was initiated. Subsequent decay curves corresponding to OA-CdSe QDs + n eq MS with the quencher were fit according to the lifetime parameters calculated for the pre-quenching sample and an additional, floating parameter used to examine the quenching process.

Photoluminescence Quenching

QD samples were placed in a quartz cuvette with a 1 cm path length for all absorption and emission characterization. A PerkinElmer Lambda 950 UV/Vis/NIR spectrophotometer was used to record all absorbance spectra. Following all syntheses, the OA-CdSe QD concentration was calculated using the first excitonic absorbance transition as described by Yu, et. al.[3] Photoluminescence (PL) spectra were measured with a Horiba FluoroMax + SpectroFluorometer. OA-CdSe QDs was treated with 20 equivalents of Meerwein's Salt (MS) in dichloromethane. This was then followed by the addition of varying equivalents of cluster in each fluorometer cuvette.

Stern-Volmer Quenching Constant[4]

The Stern-Volmer quenching constant (K_{sv}) was determined from steady-state photoluminescence quenching measurements using Stern- Volmer relationship:

$$\frac{I_0}{I} = 1 + Ksv[Q] \quad (1)$$

Where I_0 is the photoluminescence intensity of the QDs in the absence of quencher, I is in the photoluminescence intensity in the presence of the quencher and [Q] is the quencher concentration.

The ratio of $\frac{I_0}{I}$ was calculated and plotted as a function of quencher concentration. The Stern-Volmer quenching constant K_{sv} was obtained from the slope of the linear fit to the Stern-Volmer plot.

Hole transfer efficiencies calculations

Hole transfer efficiency derived from PL lifetimes can be calculated by taking the ratio of the integrated area under the time-resolved PL decay curves with and without the cluster according to:[2]

$$E_{HT}^{lifetimes} = 1 - \frac{\int PL(QDs + MS + V_6O_7^{1-})}{\int PL(QDs + MS)} \qquad (1)$$

A separate triple exponential tail fit, independent of the number of acceptors estimation, was utilized for this process to account for all photons emitted beyond the 12 ns scale utilized for the iterative reconvolution.

And the hole transfer efficiency based in steady-state (quenching) is defined as:

$$E_{HT}^{quenching} = 1 - \frac{I}{I_0} \qquad (2)$$

PL decay dynamics analysis

Intrinsic QD dynamics were obtained by fitting the kinetics of QDs + n eq MS (*n =20*) without clusters to yield the amplitudes A_i and time constants τ_{QDi} ($\tau_{QDi} = 1/k_{QDi}$). The kinetics of the QDs + n eq MS with various $V_6O_7^{1-}$ cluster concentrations were globally fit, holding the amplitudes A_i and rate constants k_{QDi} of the intrinsic electron relaxation constant. To account for the HT process (assumed to be mono-exponential), the number of acceptors *m* was allowed to vary while the rate constant k_{HTj} was held constant, as it denotes the rate for a 1:1 cluster to QD + n eq MS system. The intrinsic QD + n eq MS dynamics and the HT process are treated independently, leaving $\sum_i A_i = 1$. The lifetimes reported are amplitude-averaged lifetimes $\langle\tau_{QD}\rangle = \frac{\sum_i A_i \tau_{QDi}}{\sum_i A_i}$, appropriate for the Stern-Volmer analysis performed.

Number of molecular acceptors estimation

We estimate the number of molecular acceptors (POV- alkoxide) interacting with the QDs by fitting the PL lifetime data to a model proposed by Tachiya et al[5]. This model assumes a Poissonian distribution of quencher per chromophore given by:

$$f_n = \frac{m^n}{n!} e^{-m} \quad (1)$$

where f_n is the probability of a given QD being quenched by n $V_6O_7^{1-}$ molecules and $m=\langle n\rangle$[4-7]. The total rate of hole transfer is the product of the effective number of POV-alkoxides per QD (n) and the hole transfer rate for one cluster per QD:

$$k_{HT-total} = nk_{HT} \quad (2)$$

Therefore, the PL decay of excited QDs as a function of time is represented by the equation:

$$I(t) = I_0(\sum_i A_i e^{-k_{QD_i}t})(\sum_{n=0}^{\infty} f_n e^{-nk_{HT}t}) \quad (3)$$

I_0 is a normalization constant corresponding to the initial concentration of excited QDs and A_i is the amplitude of the ith component of the QD PL decay without a cluster. Mathematically can be demonstrated that the sum corresponding to the hole transfer process (right sum in equation 3) can be simplified to:[5]

$$I(t) = I_0(\sum_i A_i e^{-k_{QD_i}t})[e^{(-m+me^{-k_{HT}t})}] \quad (4)$$

An iterative reconvolution process was utilized to produce fits to the prior equations, where:

$$I(t) = [I_{Actual}(t) * IRF(t - \Delta t)] + B \quad (5)$$

I_{Actual} corresponds to the QD PL decay before convolution with the IRF to produce the measured signal and therefore contains the desired photophysics. This was first utilized to obtain the intrinsic QD decay dynamics, and subsequently within the global fit according to equation 4 to estimate the number of molecular acceptors.

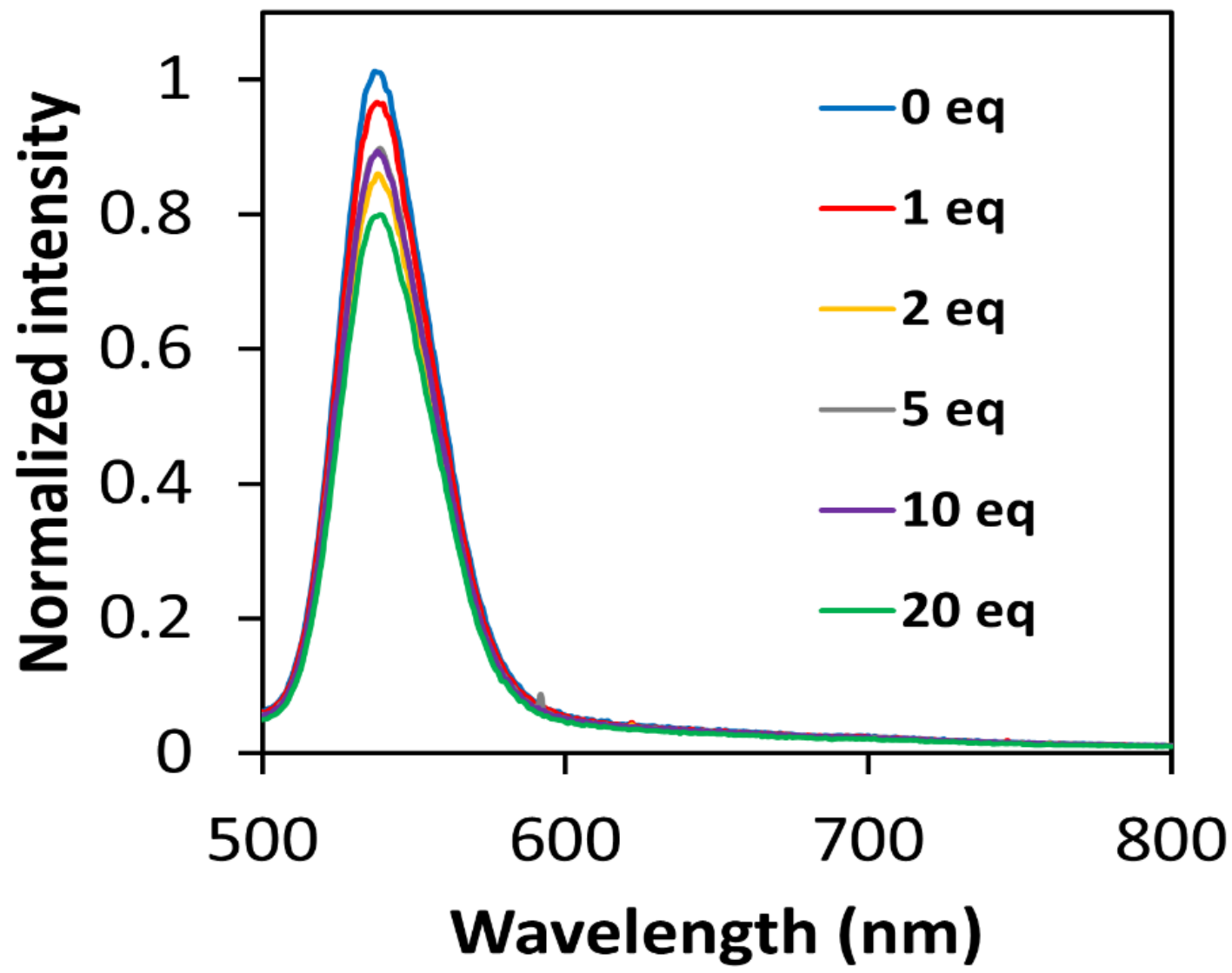

Figure S1. Normalized PL intensity of $[V_6O_7(OCH_2CH_3)_{12}]^{1-}$ + OA-CdSe.

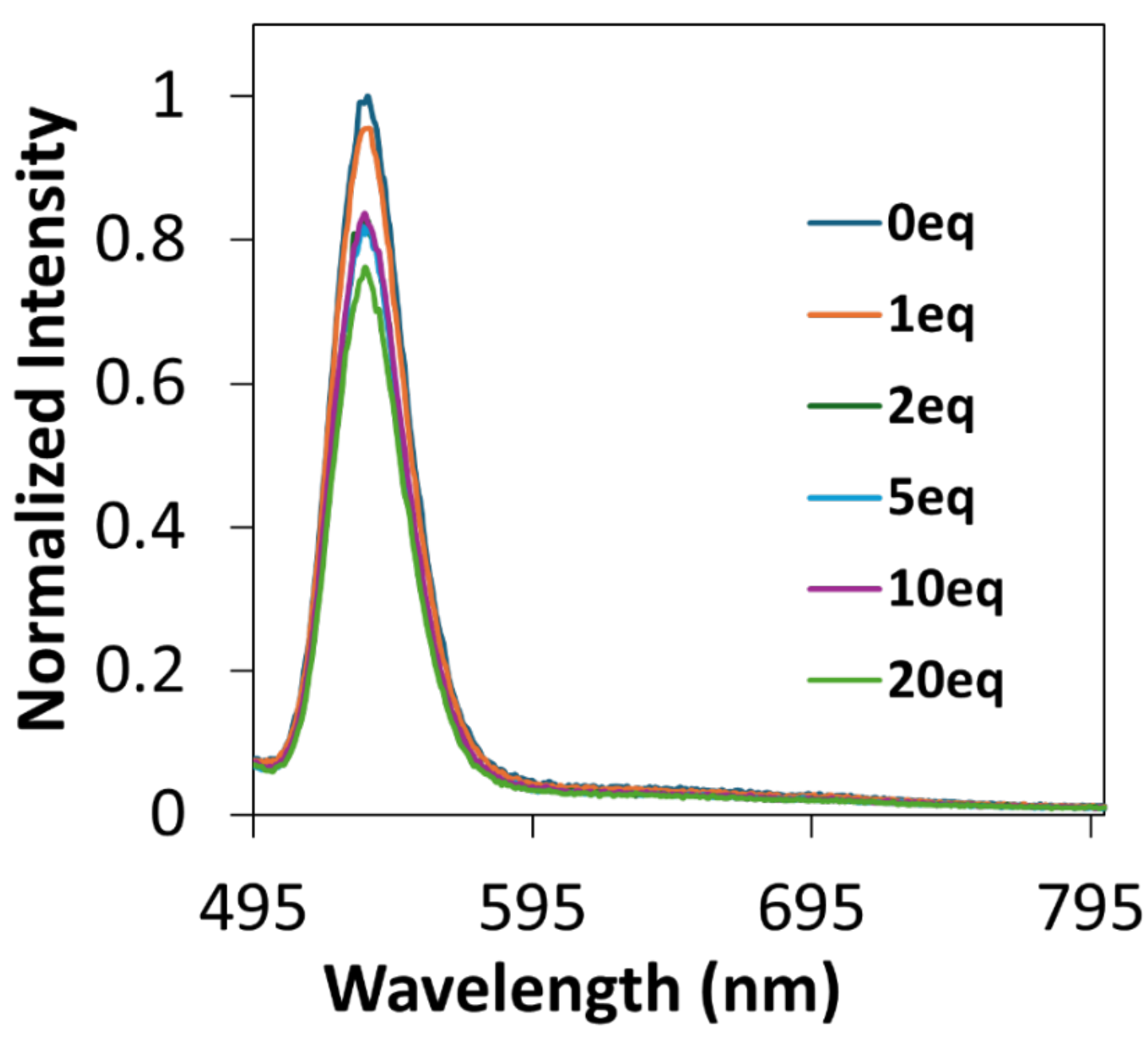

Figure S2. Normalized PL intensity of $[V_6O_7(OCH_2CH_3)_9TRIOL^{Me}]^{1-}$ + OA-CdSe.

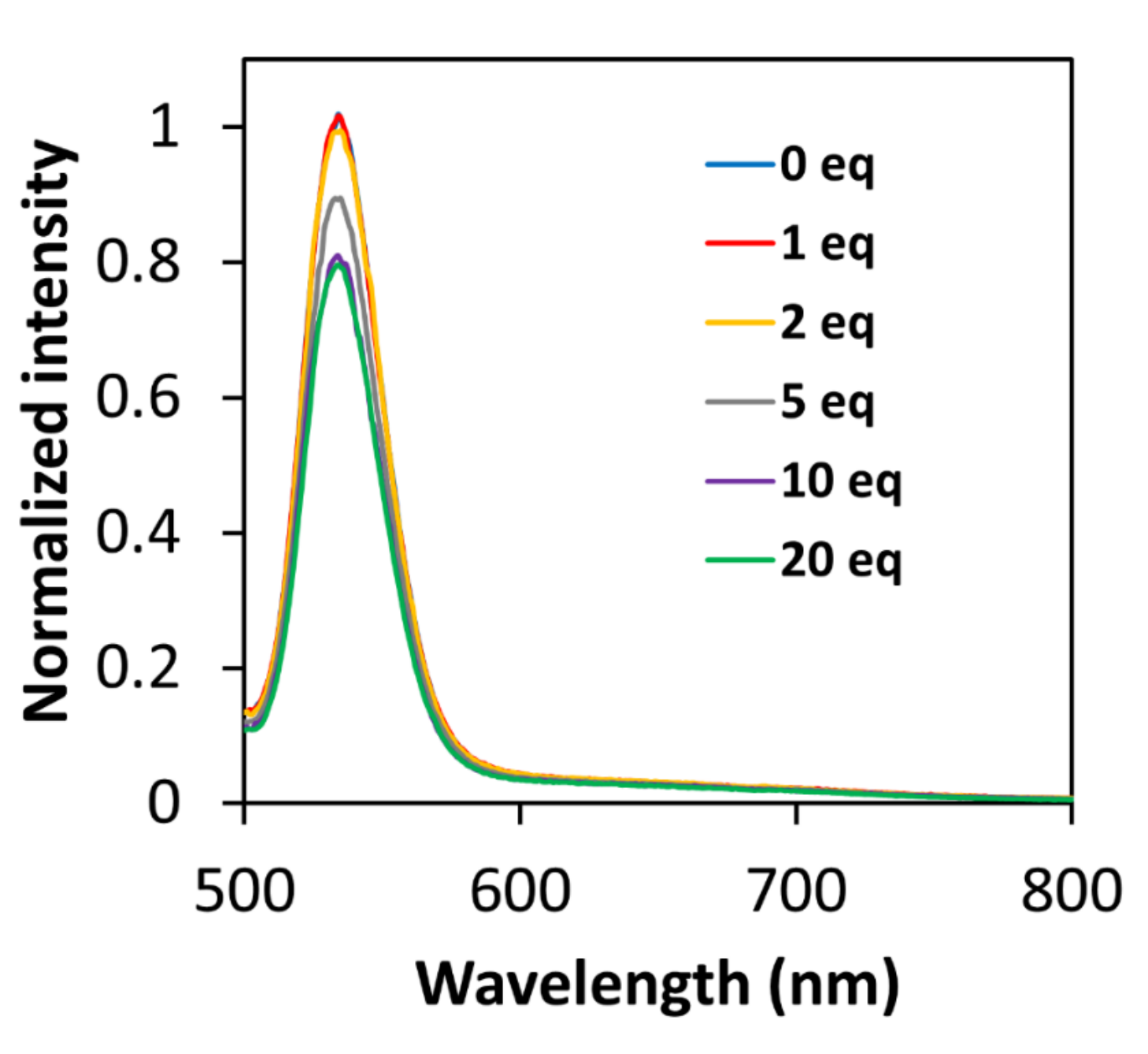

Figure S3. Normalized PL intensity of $[V_6O_7(OCH_2CH_3)_9TRIOL^{Py}]^{1-}$ + OA-CdSe.

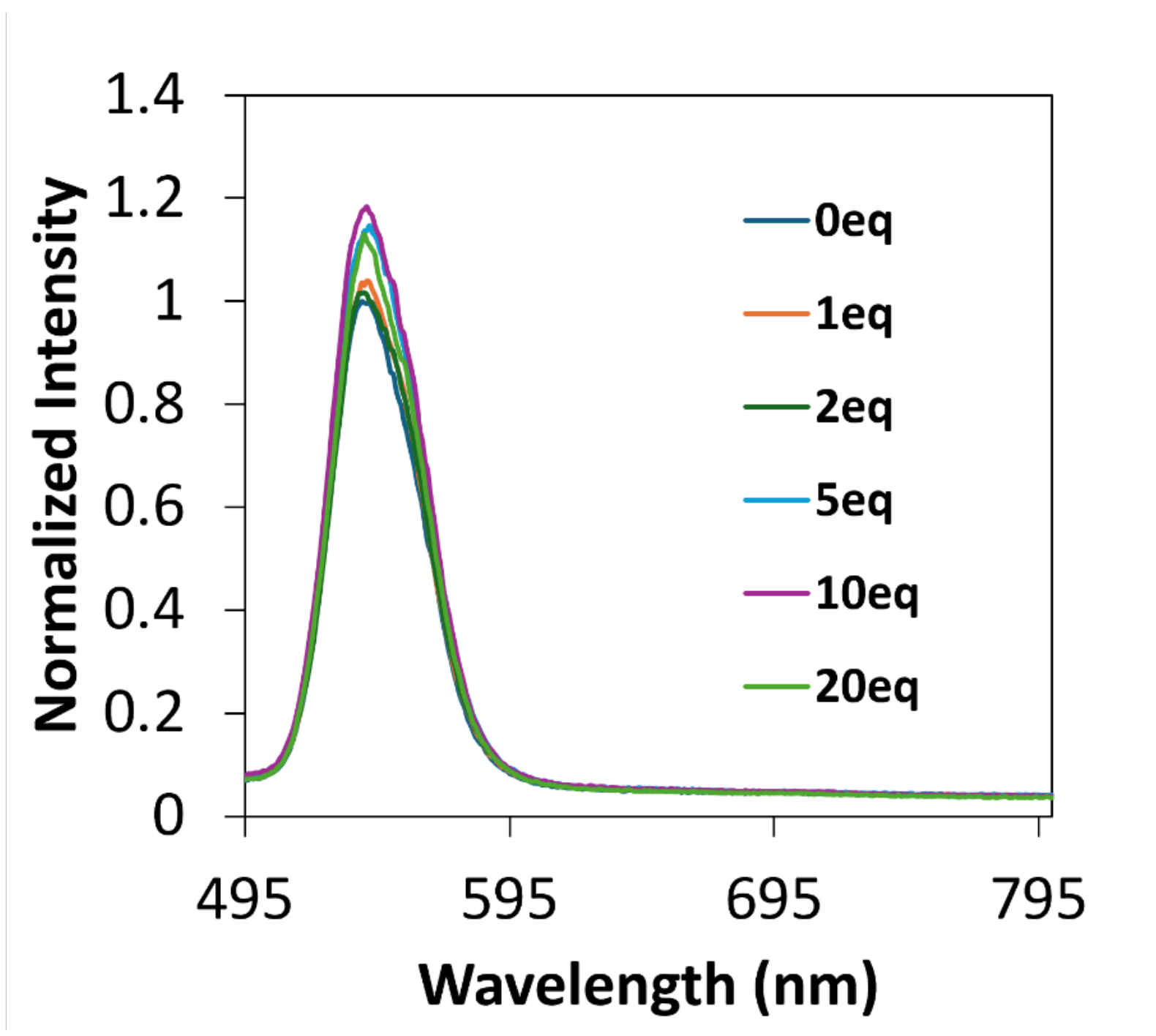

Figure S4. Normalized PL intensity of free 4-methyl pyridine + 10 eq MS treated OA-CdSe.

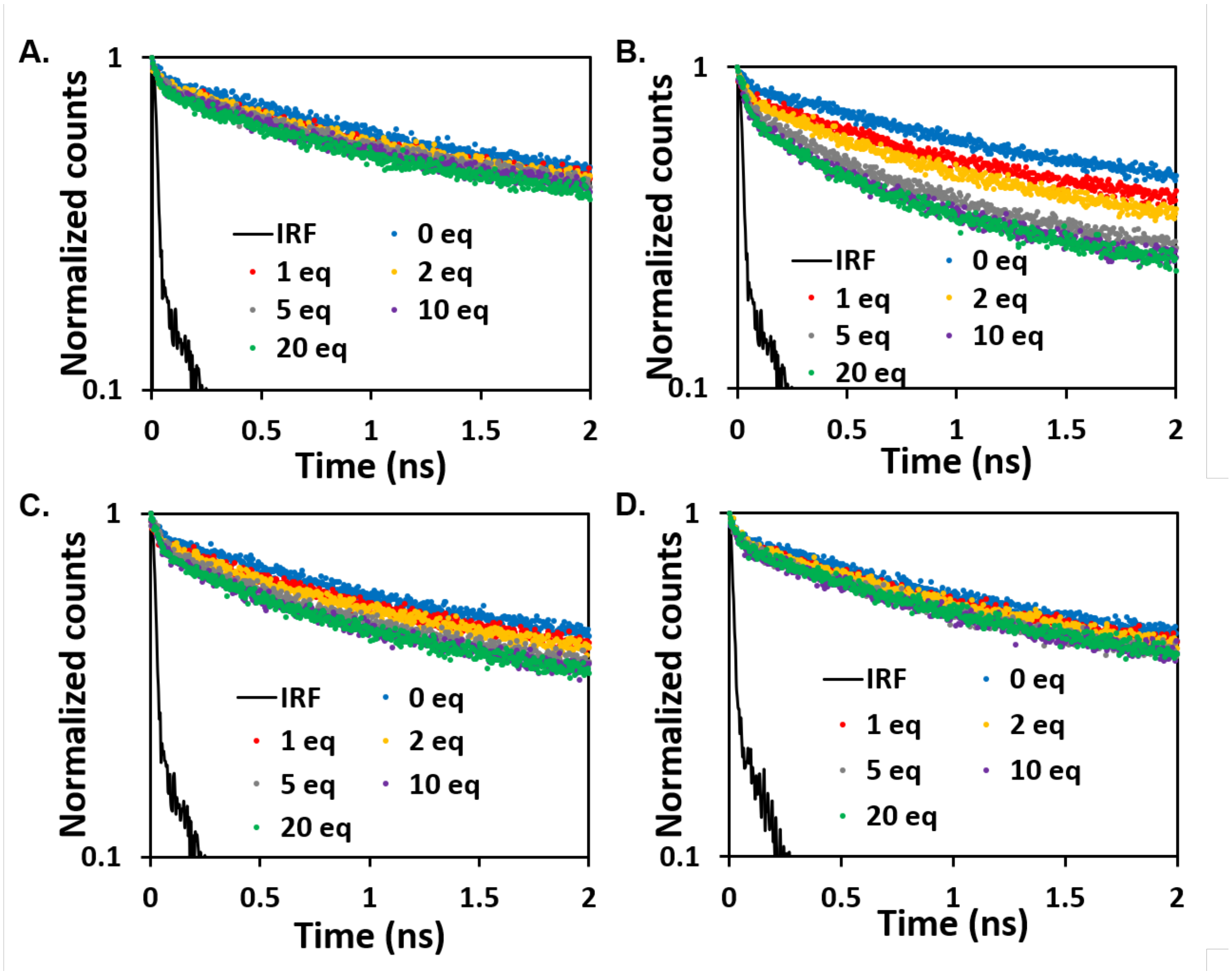

Figure S5. Short timescale TCSPC traces for OA-CdSe QDs + 20 eq MS + (**A**) $[V_6O_7(OCH_2CH_3)_{12}]^{1-}$ (**B**) $V_6O_7(OCH_3)_{12}{}^{1-}$ (**C**) $[V_6O_7(OCH_2CH_3)_9TRIOL^{Me}]^{1-}$ and (**D**) $[V_6O_7(OCH_2CH_3)_9TRIOL^{Py}]^{1-}$.

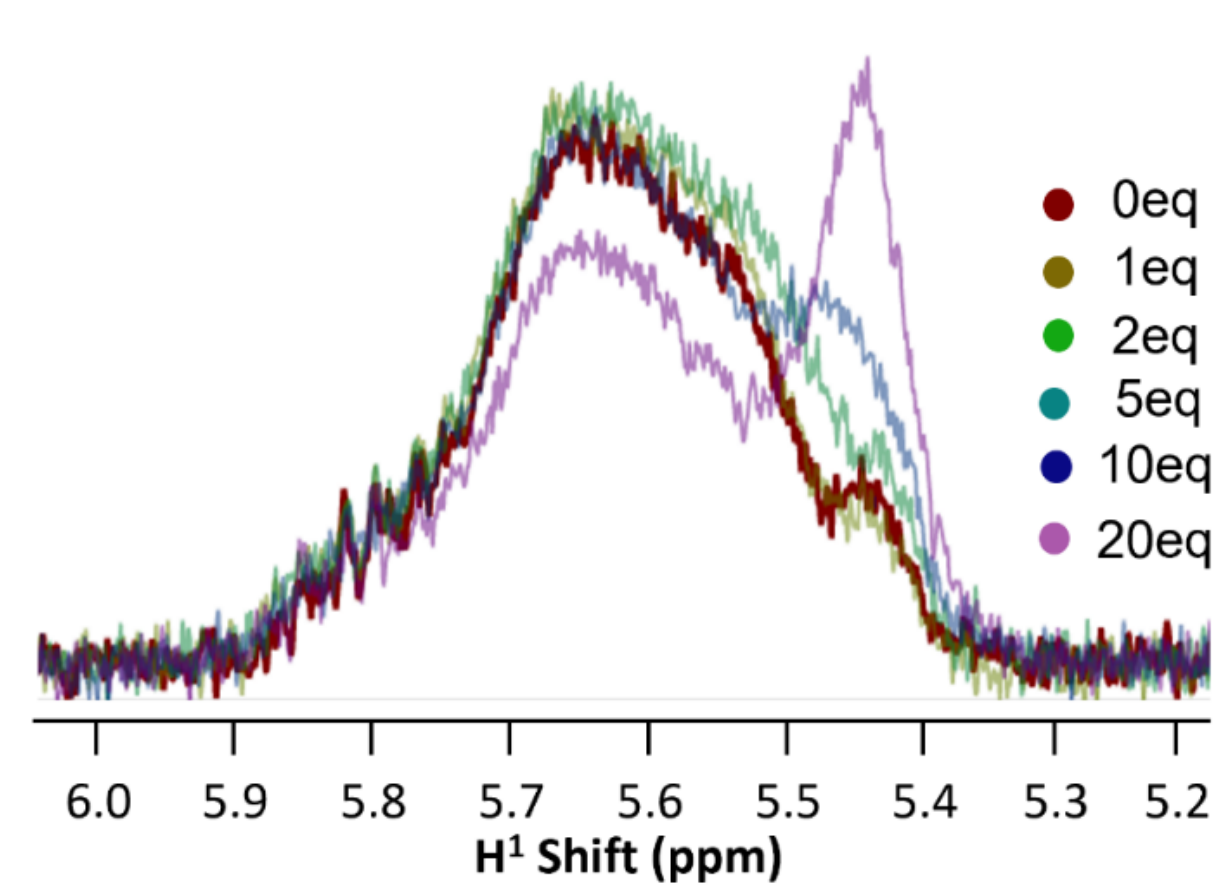

Figure S6. ^{1}H NMR spectra of OA-CdSe QDs with 0-20 eq MS in toluene-d_8.

Shown is the alkene region of the full spectrum, which displays both bound OA (5.6 ppm) and free methyl oleate (5.4 ppm). Analysis of the alkene signal from OA allows for the ability to distinguish between ligands bound to the nanocrystal surface and those that are free based on their relative broadening and chemical shift in deuterated aromatic solvents. This is predominantly due to the differences of chemical environment for bound and free ligand species. OA bound to the QD surface tumbles more slowly through solution as compared to free methyl oleate, which tumbles at a faster rate and therefore exhibits signal sharpening in addition to an aromatic solvent-induced shift in toluene-d_8.[7, 8]

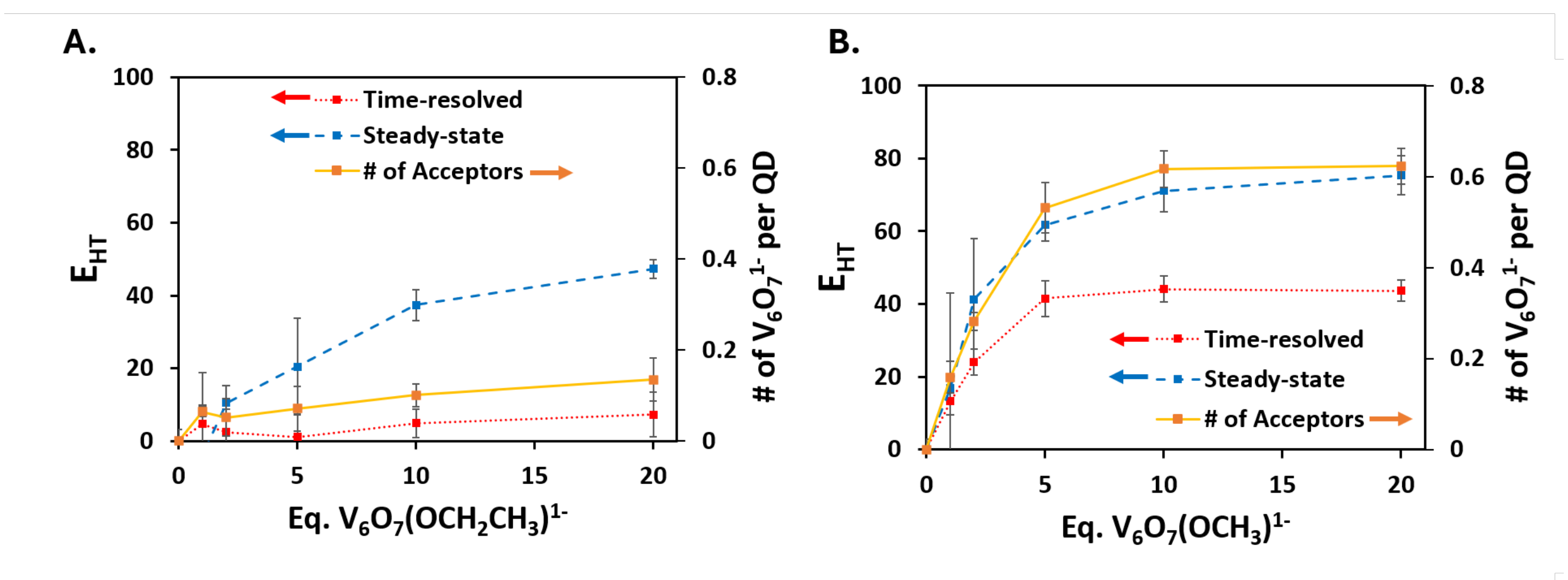

Figure S7. Comparison of HT efficiencies for OA-CdSe QDs + 20 eq MS + (**A**) $[V_6O_7(OCH_2CH_3)_{12}]^{1-}$ and (**B**) $[V_6O_7(OCH_3)_{12}]^{1-}$.

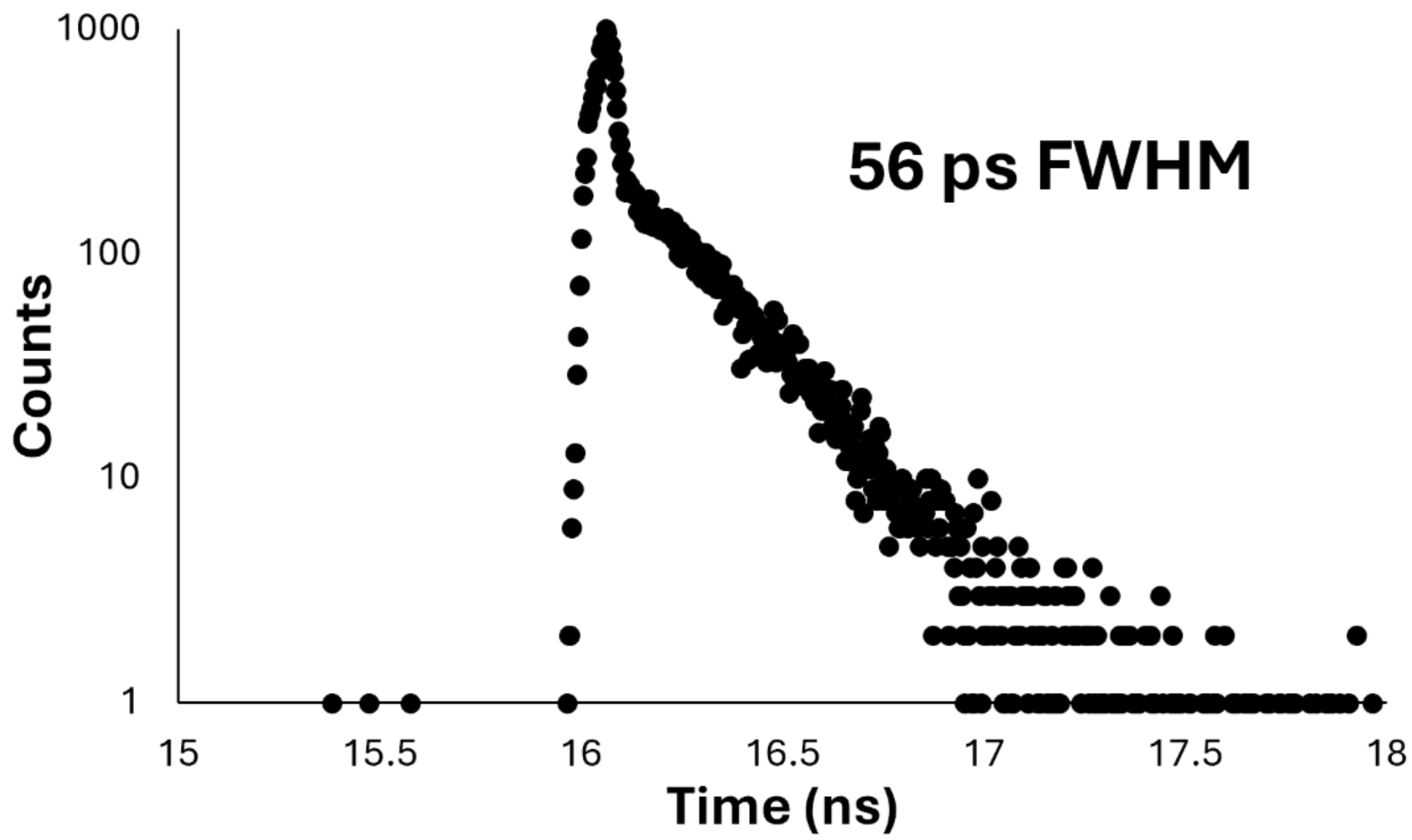

Figure S8. Instrument response function for TCSPC measurements.

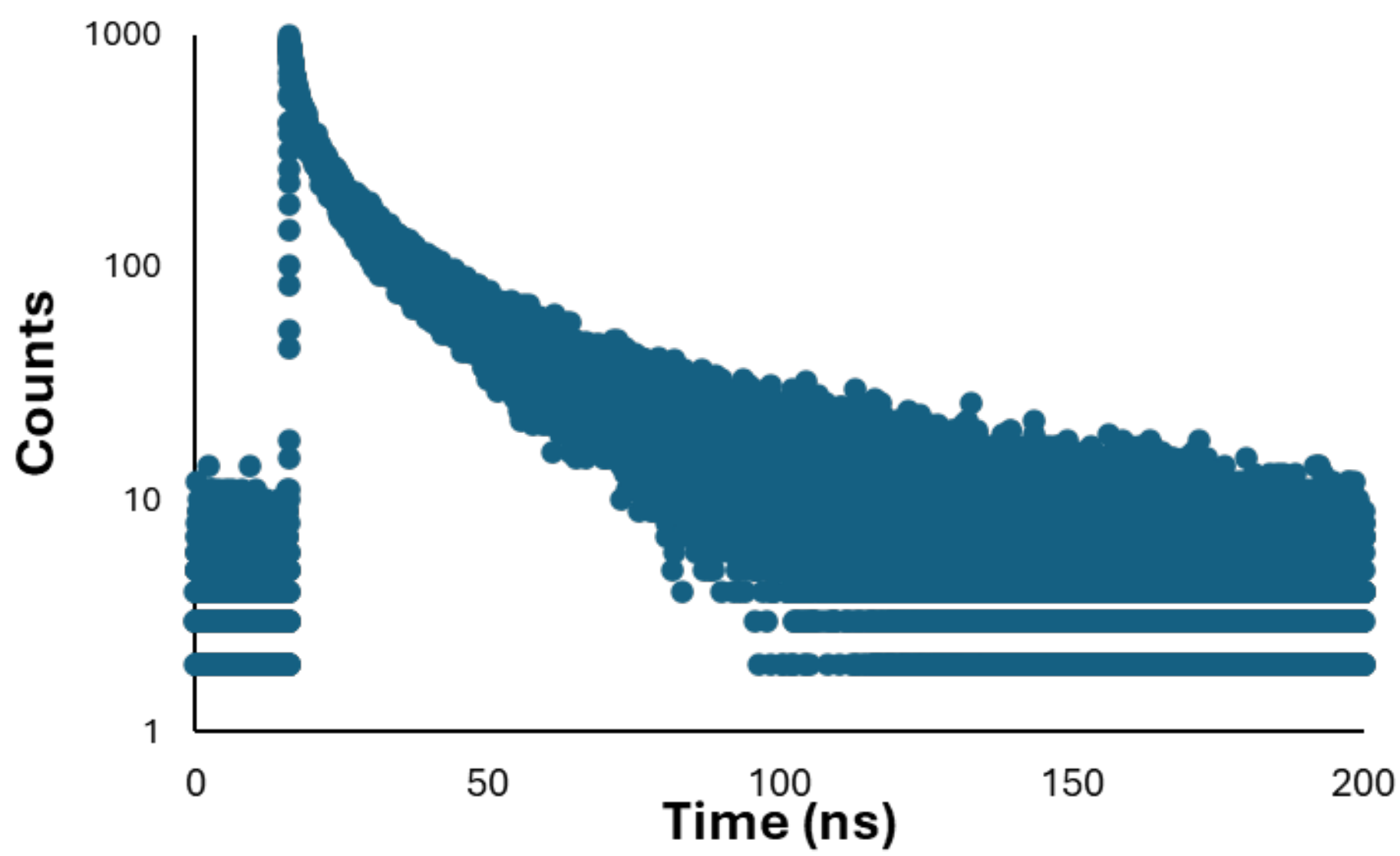

Figure S9. Example of untruncated TCSPC curve for OA-CdSe QDs + 20 eq MS.

Table S1. Summary of TCSPC Fitting Parameters for OA-CdSe QDs + 20 eq MS + $[V_6O_7(OCH_2CH_3)_{12}]^{1-}$

Fitting parameters for TCSPC lifetime measurements of OA-CdSe QDs + 20 eq MS with $[V_6O_7(OCH_2CH_3)_{12}]^{1-}$ with an example of associated fits, prior to averaging, shown below.

**Intrinsic Relaxation Kinetics**				**Hole Transfer Kinetics**
τ_{QD1}<br>A_1	τ_{QD2}<br>A_2	τ_{QD3}<br>A_3	$\langle\tau_{QD}\rangle$*	τ_{1HT}<br>B_1
0.09 ± 0.00 ns<br>*64.8 ± 1.1 %*	1.38 ± 0.03 ns<br>*16.5 ± 0.5%*	12.22 ± 0.29 ns<br>*18.6 ± 0.6 %*	2.56 ± 0.04 ns	0.07 ± 0.02 ns<br>*100 %*

**Hole Transfer Kinetics with Various Equivalents of POV-alkoxide**		
**# Equiv. $V_6O_7^{1-}$**	**# acceptors (m)**	τ_{nHT}#
**1**	0.06 ± 0.01	1.06 ± 0.37 ns
**2**	0.05 ± 0.04	1.33 ± 1.14 ns
**5**	0.07 ± 0.05	0.95 ± 0.71 ns
**10**	0.10 ± 0.03	0.68 ± 0.27 ns
**20**	0.14 ± 0.05	0.51 ± 0.23 ns

*Amplitude-weighted average lifetime $\langle\tau_{QD}\rangle = \frac{\sum_i A_i \tau_{QDi}}{\sum_i A_i}$. #Hole transfer lifetime ($\frac{1}{k_{nj}} = \frac{n}{k_{1HT}}$). All errors are estimated by the standard deviation of multiple measurements.

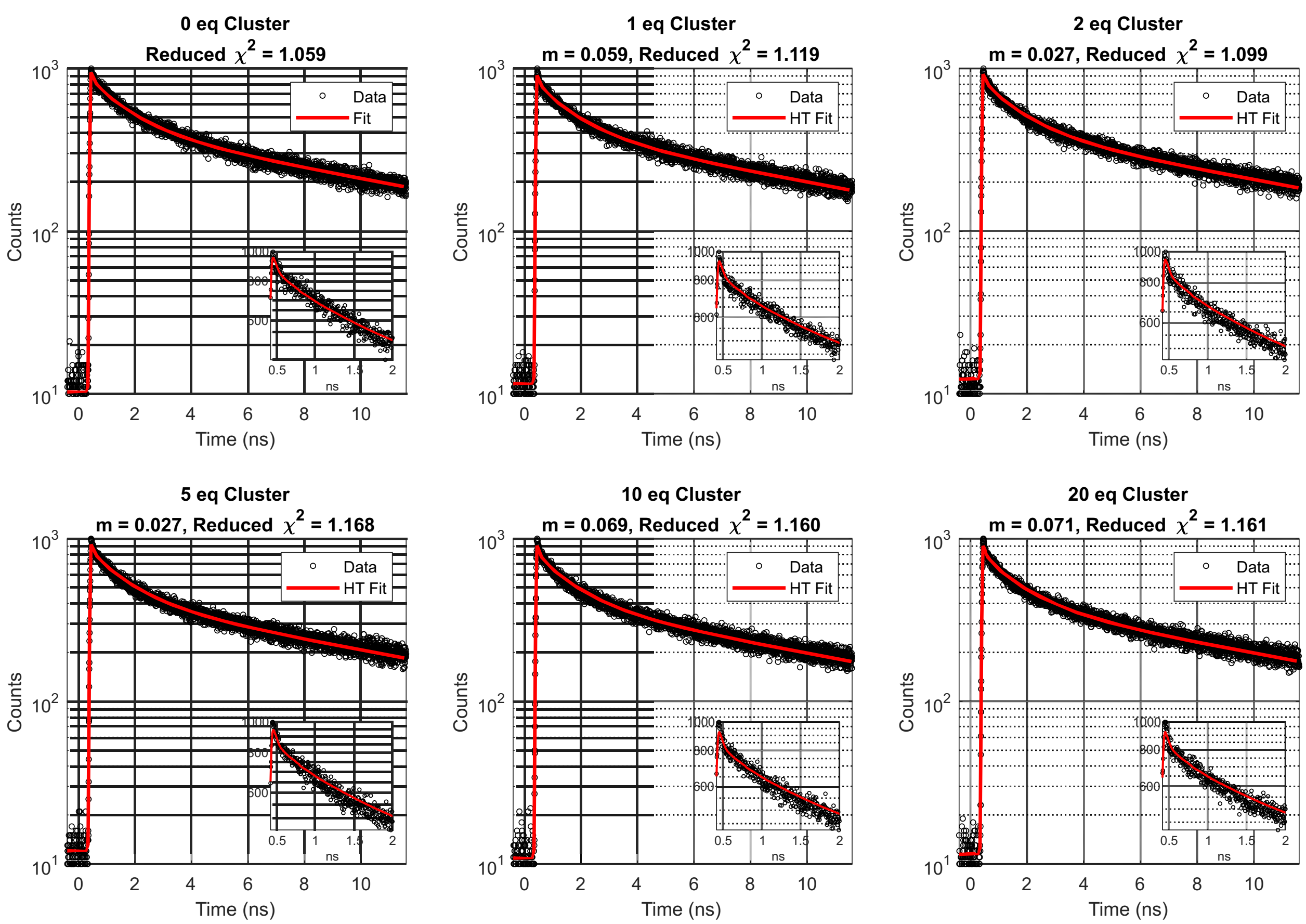

Table S2. Summary of TCSPC Fitting Parameters for OA-CdSe QDs + 20 eq MS + $[V_6O_7(OCH_3)_{12}]^{1-}$.

Fitting parameters for TCSPC lifetime measurements of OA-CdSe QDs + 20 eq MS with $[V_6O_7(OCH_3)_{12}]^{1-}$ with an example of associated fits, prior to averaging, shown below.

Intrinsic Relaxation Kinetics				Hole Transfer Kinetics
τ_{QD1} A_1	τ_{QD2} A_2	τ_{QD3} A_3	$\langle\tau_{QD}\rangle^*$	τ_{1HT} B_1
0.08 ± 0.00 ns *65.1 ± 1.2 %*	1.30 ± 0.01 ns *16.3 ± 0.6%*	11.56 ± 0.07 ns *18.6 ± 0.6 %*	2.42 ± 0.07 ns	0.30 ± 0.04 ns *100 %*

Hole Transfer Kinetics with Various Equivalents of POV-alkoxide		
**# Equiv. $V_6O_7^{1-}$**	**# acceptors (m)**	**$\tau_{nHT}^{\#}$**
**1**	0.16 ± 0.04	1.92 ± 0.49 ns
**2**	0.28 ± 0.02	1.08 ± 0.16 ns
**5**	0.53 ± 0.06	0.57 ± 0.09 ns
**10**	0.62 ± 0.04	0.49 ± 0.07 ns
**20**	0.62 ± 0.04	0.49 ± 0.07 ns

*Amplitude-weighted average lifetime $\langle\tau_{QD}\rangle = \frac{\sum_i A_i \tau_{QDi}}{\sum_i A_i}$. #Hole transfer lifetime ($\frac{1}{k_{nj}} = \frac{n}{k_{1HT}}$). All errors are estimated by the standard deviation of multiple measurements.

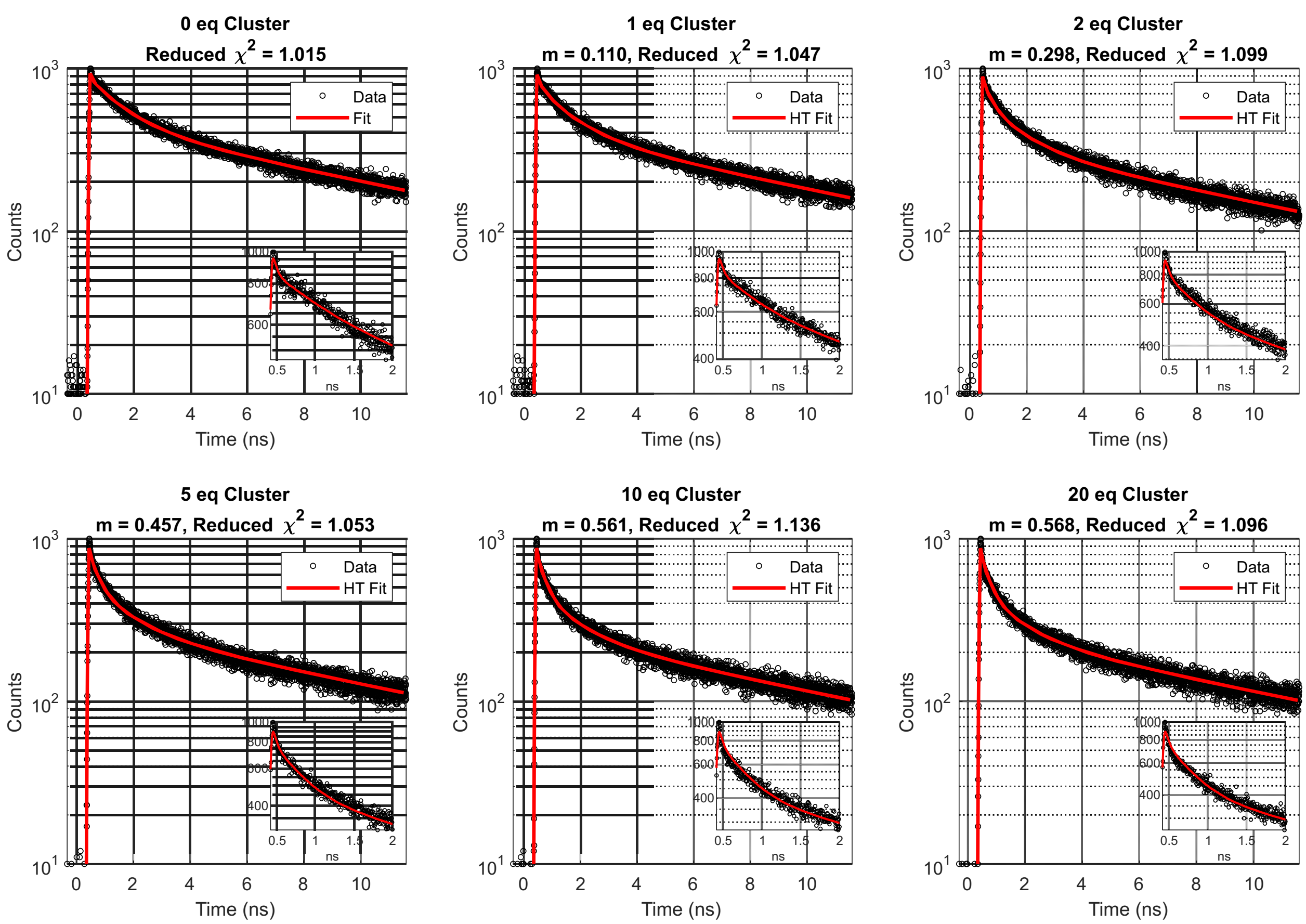

Table S1. Summary of TCSPC Fitting Parameters for OA-CdSe QDs + 20 eq MS + $[V_6O_7(OCH_2CH_3)_9TRIOL^{Me}]^{1-}$.

Fitting parameters for TCSPC lifetime measurements of OA-CdSe QDs + 20 eq MS with $[V_6O_7(OCH_2CH_3)_9TRIOL^{Me}]^{1-}$ with an example of associated fits, prior to averaging, shown below.

**Intrinsic Relaxation Kinetics**				**Hole Transfer Kinetics**
τ_{QD1}<br>A_1	τ_{QD2}<br>A_2	τ_{QD3}<br>A_3	$\langle\tau_{QD}\rangle$*	τ_{1HT}<br>B_1
0.08 ± 0.00 ns<br>*66.7 ± 0.6%*	1.32 ± 0.03 ns<br>*15.5 ± 0.3 %*	12.08 ± 0.40 ns<br>*17.8 ± 0.3 %*	2.41 ± 0.12 ns	0.09± 0.05 ns<br>*100 %*

**Hole Transfer Kinetics with Various Equivalents of POV-alkoxide**		
**# Equiv. $V_6O_7^{1-}$**	**# acceptors (m)**	τ_{nHT}#
**1**	0.04 ± 0.02	2.65 ± 1.82 ns
**2**	0.04 ± 0.02	2.59 ± 2.15 ns
**5**	0.08 ± 0.05	1.21 ± 1.01 ns
**10**	0.12 ± 0.04	0.76 ± 0.44 ns
**20**	0.13 ± 0.02	0.75 ± 0.41 ns

*Amplitude-weighted average lifetime $\langle\tau_{QD}\rangle = \frac{\sum_i A_i \tau_{QDi}}{\sum_i A_i}$. #Hole transfer lifetime ($\frac{1}{k_{nj}} = \frac{n}{k_{1HT}}$). All errors are estimated by the standard deviation of multiple measurements.

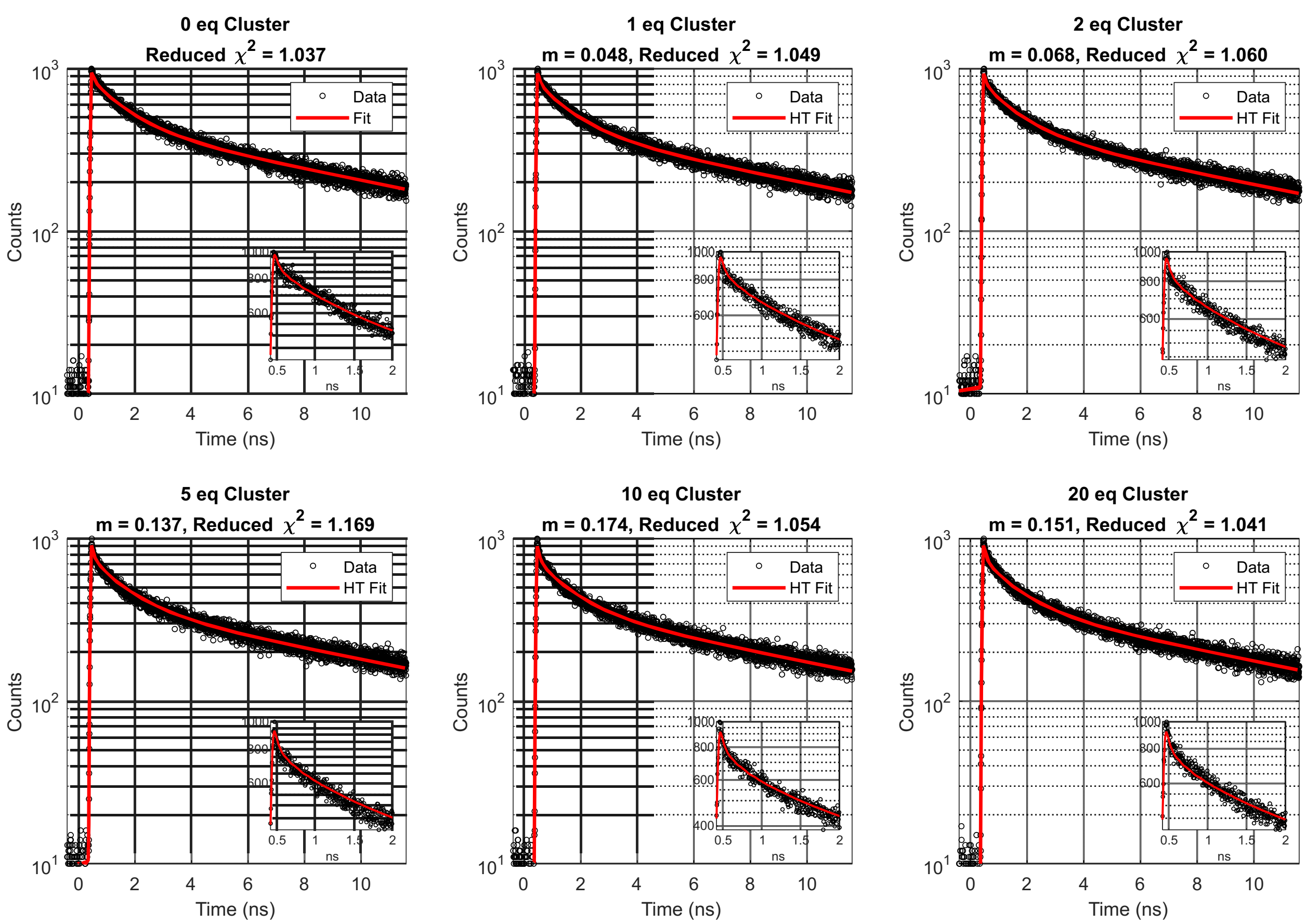

Table S2. Summary of TCSPC Fitting Parameters for OA-CdSe QDs + 20 eq MS + $[V_6O_7(OCH_2CH_3)_9TRIOL^{Py}]^{1-}$

Fitting parameters for TCSPC lifetime measurements of OA-CdSe QDs + 20 eq MS with $[V_6O_7(OCH_2CH_3)_9TRIOL^{Py}]^{1-}$ with an example of associated fits, prior to averaging, shown below.

**Intrinsic Relaxation Kinetics**				**Hole Transfer Kinetics**
τ_{QD1}<br>A_1	τ_{QD2}<br>A_2	τ_{QD3}<br>A_3	$\langle\tau_{QD}\rangle$*	τ_{1HT}<br>B_1
0.08 ± 0.00 ns<br>*67.2 ± 0.9 %*	1.25 ± 0.04 ns<br>*15.2 ± 0.3 %*	11.79 ± 0.35 ns<br>*17.6 ± 0.6 %*	2.32 ± 0.15 ns	0.71 ± 0.18 ns<br>*100 %*

**Hole Transfer Kinetics with Various Equivalents of POV-alkoxide**		
**# Equiv. $V_6O_7^{1-}$**	**# acceptors (m)**	**τ_{nHT}#**
**1**	0.06 ± 0.03	11.47 ± 6.22 ns
**2**	0.09 ± 0.06	7.73 ± 5.42 ns
**5**	0.17 ± 0.03	4.24 ± 1.36 ns
**10**	0.19 ± 0.06	3.82 ± 1.58 ns
**20**	0.17 ± 0.04	4.26 ± 1.54 ns

*Amplitude-weighted average lifetime $\langle\tau_{QD}\rangle = \frac{\sum_i A_i \tau_{QDi}}{\sum_i A_i}$. #Hole transfer lifetime ($\frac{1}{k_{nj}} = \frac{n}{k_{1HT}}$). All errors are estimated by the standard deviation of multiple measurements.

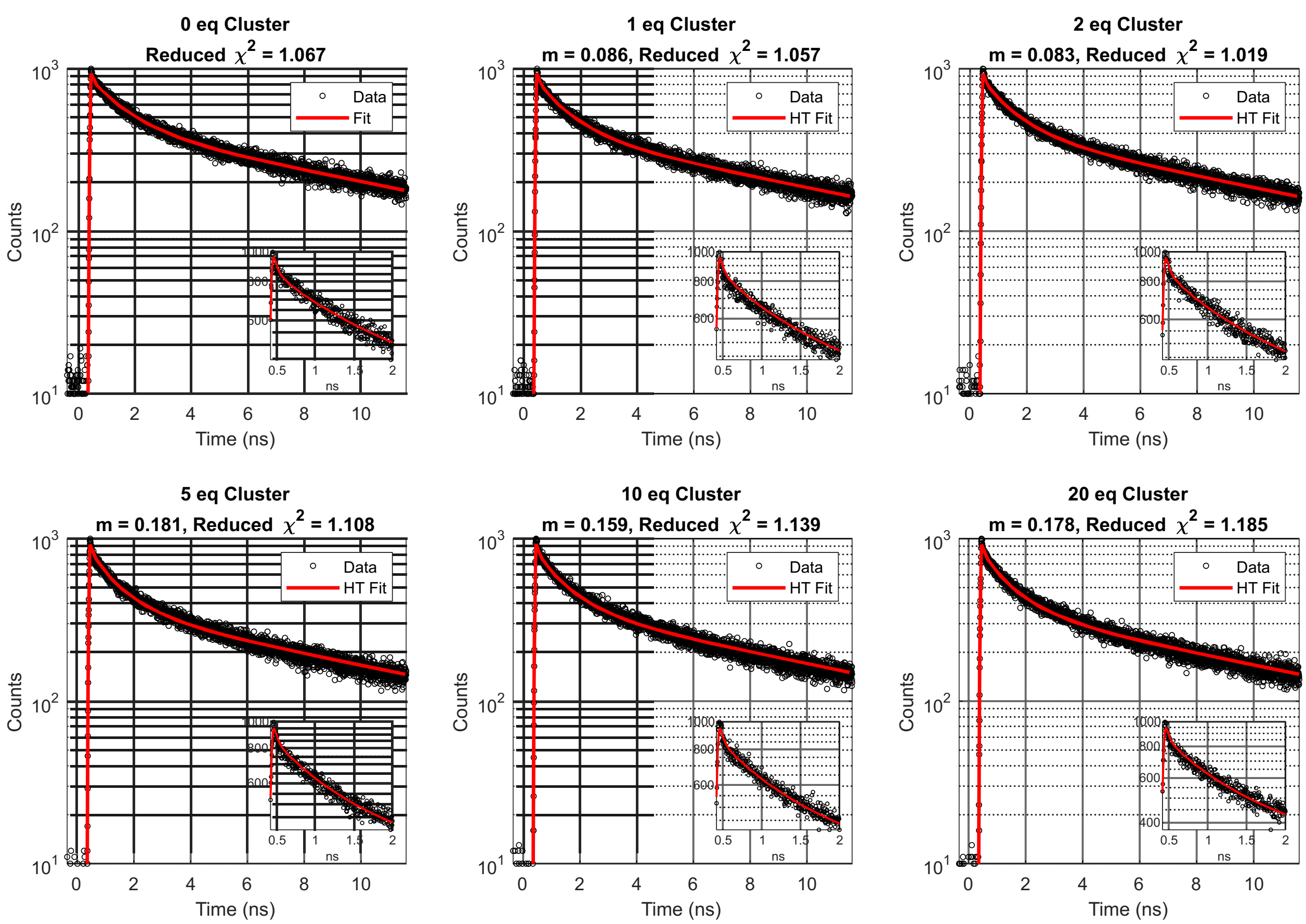

Table S3. Quantified bound, free methyl oleate, and total ligand per QD via ^{1}H NMR spectroscopy.

Quantified bound OA per QD, free methyl oleate per QD, and total ligand per QD as calculated via ^{1}H NMR peak fitting and integration in MestReNova against a ferrocene internal standard.

MS	Bound OA/QD	Free Methyl Oleate/QD	Total Ligands/ QD
**QD Only**	112.83	4.81	117.64
**1eq**	112.19	4.83	117.02
**5eq**	110.35	10.88	121.23
**10eq**	98.93	22.41	121.34
**20eq**	88.73	31.19	119.91
		**Average Ligand/QD**	119.43 ± 2

References:

(1) Peter, C. Y. M.; Schreiber, E.; Proe, K. R.; Matson, E. M. Surface ligand length influences kinetics of H-atom uptake in polyoxovanadate-alkoxide clusters. *Dalton Trans.* **2023**, *52*, 15775-15785.

(2) Schurr, B. E.; Nachtigall, O.; VanGelder, L. E.; Drappeau, J.; Brennessel, W. W.; Matson, E. M. Consequences of ligand derivatization on the electronic properties of polyoxovanadate-alkoxide clusters. *J. Coord. Chem.* **2019**, *72*, 1267-1286.

(3) Yu, W. W.; Qu, L. H.; Guo, W. Z.; Peng, X. G. Experimental determination of the extinction coefficient of CdTe, CdSe and CdS nanocrystals. *Chem. Mater.* **2004**, *16*, 560-560.

(4) Lakowicz, J. R. Quenching of Fluorescence. In *Principles of Fluorescence Spectroscopy*, Springer US, 1983; pp 257-301.

(5) Sadhu, S.; Tachiya, M.; Patra, A. A Stochastic Model for Energy Transfer from CdS Quantum Dots/Rods (Donors) to Nile Red Dye (Acceptors). *J. Phys. Chem. C.* **2010**, *114*, 2842-2842.

(6) Cogan, N. M. B.; McClelland, K. P.; Peter, C. Y. M.; Rodríguez, C. C.; Fertig, A. A.; Amin, M.; Brennessel, W. W.; Krauss, T. D.; Matson, E. M. Efficient Hole Transfer from CdSe Quantum Dots Enabled by Oxygen-Deficient Polyoxovanadate-Alkoxide Clusters. *Nano Lett.* **2023**, *23*, 10221-10227.

(7) Peter, C. Y. M.; Rodríguez, C. C.; Gorski, H. N.; Phinney, E. O.; Krauss, T. D.; Matson, E. M. Modulating Hole Transfer from CdSe Quantum Dots by Manipulating the Surface Ligand Density. *Nano Lett.* **2025**, *25*, 8993-8998.

(8) Knauf, R. R.; Lennox, J. C.; Dempsey, J. L. Quantifying Ligand Exchange Reactions at CdSe Nanocrystal Surfaces. *Chem. Mater.* **2016**, *28*, 4762-4770.
